# Loss of function of 1-FEH IIb has more impact on post-harvest inulin degradation in *Cichorium intybus* than copy number variation of its close paralog 1-FEH IIa

**DOI:** 10.3389/fpls.2015.00455

**Published:** 2015-06-23

**Authors:** Nicolas Dauchot, Pierre Raulier, Olivier Maudoux, Christine Notté, Xavier Draye, Pierre Van Cutsem

**Affiliations:** ^1^Research Unit in Plant Biology, University of NamurNamur, Belgium; ^2^Earth and Life Institute, Université Catholique de LouvainLouvain-la-Neuve, Belgium; ^3^Chicoline-CosucraWarcoing, Belgium

**Keywords:** association study, CNV, chicory, fructan, fructan exohydrolases, FEH, GH32, inulin

## Abstract

**Key Message:** The loss of mini-exon 2 in the 1-FEH IIb glycosyl-hydrolase results in a putative non-functional allele. This loss of function has a strong impact on the susceptibility to post-harvest inulin depolymerization. Significant variation of copy number was identified in its close paralog 1-FEH IIa, but no quantitative effect of copy number on carbohydrates-related phenotypes was detected.

Inulin polyfructan is the second most abundant storage carbohydrate in flowering plants. After harvest, it is depolymerized by fructan exohydrolases (FEHs) as an adaptive response to end-season cold temperatures. In chicory, the intensity of this depolymerization differs between cultivars but also between individuals within a cultivar. Regarding this phenotypic variability, we recently identified statistically significant associations between inulin degradation and genetic polymorphisms located in three FEHs. We present here new results of a systematic analysis of copy number variation (CNV) in five key members of the chicory (*Cichorium intybus*) GH32 multigenic family, including three FEH genes and the two inulin biosynthesis genes: 1-SST and 1-FFT. qPCR analysis identified a significant variability of relative copy number only in the 1-FEH IIa gene. However, this CNV had no quantitative effect. Instead, cloning of the full length gDNA of a close paralogous sequence (1-FEH IIb) identified a 1028 bp deletion in lines less susceptible to post-harvest inulin depolymerization. This region comprises a 9 bp mini-exon containing one of the three conserved residues of the active site. This results in a putative non-functional 1-FEH IIb allele and an observed lower inulin depolymerization. Extensive genotyping confirmed that the loss of mini-exon 2 in 1-FEH IIb and the previously identified 47 bp duplication located in the 3′UTR of 1-FEH IIa belong to a single haplotype, both being statistically associated with reduced susceptibility to post-harvest inulin depolymerization. Emergence of these haplotypes is discussed.

## Introduction

Industrial chicory is the main commercial source of inulin, a linear fructose polymer used by the agro-industry as texturizer, fat substitute (Mendoza et al., 2001; Keenan et al., 2014; Karimi et al., 2015) or low calories sweetener. Inulin is also used by pharmaceutical industries for its health promoting properties (Gibson et al., 2006; Meyer and Stasse-Wolthuis, 2009). In chicory, like in most Asteraceae (Hendry, 1993), inulin is the main reserve carbohydrate. It is accumulated in the taproot during growing season and it is hydrolyzed by FEHs as an adaptive response to the exposure to end-season cold temperatures (Dauchot et al., 2014).

As first proposed in 1968 in *Helianthus tuberosus* (Edelman and Jefford, 1968), inulin synthesis results from the sequential action of the enzyme sucrose:sucrose 1-fructosyltransferase (1-SST) and fructan:fructan 1-fructosyltransferase (1-FFT). The 1-SST enzyme initiates inulin synthesis by transferring a fructose moiety from a sucrose donor molecule (GF) to a second sucrose molecule, which acts as fructose acceptor, to produce 1-kestotriose (GFF) and free glucose (G). This 1-kestotriose molecule is then used as fructose donor by the enzyme fructan:fructan 1-fructosyltransferase (1-FFT) to elongate inulin molecules (GFFn+1), releasing free sucrose (GF). Several years later, we were able to isolate the corresponding 1-FFT and 1-SST coding sequences from chicory (De Halleux and Van Cutsem, 1997). The coding sequences of chicory 1-FEH I, 1-FEH IIa, and 1-FEH IIb inulin hydrolyzing genes were isolated short after (Van den Ende et al., 2000, 2001).

In chicory, inulin chain length varies between 10 and up to 60 fructosyl units. Average inulin chain length varies along the season. At the end of the growing season, inulin is hydrolyzed to help facing winter cold temperatures, resulting in an increase of free sugars (sucrose, glucose, and fructose) and a global shortening of inulin chain length. Since chicory 1-SST and FEH genes are reported to be mainly regulated at the transcriptional level (Michiels et al., 2004), the reduction of the average inulin chain length and parallel increase of free sugars is believed to result from a reduction of 1-SST transcription and a parallel increase of the transcription of genes coding for inulin hydrolyzing enzymes (van Arkel et al., 2012), namely FEHs, while the expression of 1-FFT remains constant (Van Laere and Van den Ende, 2002).

This adaptive response to cold seriously interferes with the industrial inulin extraction process because roots start to accumulate free sugars, which negatively affects the inulin extraction process and final yield. The average size reduction of the inulin molecules also modifies their physico-chemical properties (Mensink et al., 2015) and commercial value.

Inulin chain length differs between species (Itaya et al., 2007). While the inter-specific variability of inulin chain length is believed to result from a difference of activity of the enzyme 1-FFT (Hellwege et al., 1998), the molecular bases of the high intra-specific variation of inulin chain length observed in industrial chicory cultivars (Amaducci and Pritoni, 1998; Wilson et al., 2004; Monti et al., 2005) are still unknown.

In this context, we sampled the existing phenotypic diversity observed between and within industrial chicory cultivars to identify the genetic bases responsible for the observed differences of susceptibility to post-harvest inulin depolymerization. In a previous study (Dauchot et al., 2014), we identified SNP polymorphisms statistically associated with these differences of susceptibility. These SNPs were located within a single subgroup of the GH32 multigenic family, namely the FEHs 1-FEH I, 1-FEH IIa, and 1-FEH IIb, a class of enzymes that specifically degrades inulin (Van den Ende et al., 2000, 2001). To further investigate the genetic bases of the observed phenotypic plasticity, we decided to study CNV of these three FEHs and of two closely related GH32 enzymes involved in inulin biosynthesis (1-SST and 1-FFT).

Copy number variation is extensively studied in humans and have been associated with several diseases (Almal and Padh, 2011). CNV have only recently been recognized as responsible for the high phenotypic plasticity observed in domestic dog (Alvarez and Akey, 2011; Nicholas et al., 2011) and other domestic animal species (Clop et al., 2012). In crop species, CNV started not long ago to be the subject of a growing interest in the scientific community (Springer et al., 2009; Knox et al., 2010). CNV is a spontaneous process whose rate could exceed the rate of single nucleotide polymorphisms by two orders of magnitude, as observed in *Caenorhabditis* (Lipinski et al., 2011). In *Arabidopsis*, CNV has been observed over immediate family generational scales (DeBolt, 2010). This author highlighted a putative effect of the environment on CNV and also pointed out that tandem-duplicated genes were common in CNV events. The sequence homology (94%) between the 1-FEH IIa (AJ295033) and 1-FEH IIb (AJ295034) genes coupled to their physical proximity (Cadalen et al., 2010) could favor CNV appearance in this multigenic family and result in the observed phenotypic variability of carbohydrate content and properties in chicory after harvest.

Here we identified the presence of CNV in the 1-FEH IIa gene. Despite putative functional relevance of these results, statistical tests confirmed that these CNV were not associated to quantitative variations of susceptibility to post-harvest inulin-depolymerization. On the other hand, we were also able to identify a loss-of-function allele in a close paralog (1-FEH IIb) resulting from the loss of one of the three amino acids present at the active site of the enzyme. This allele was associated with a reduced susceptibility to post-harvest inulin depolymerization.

## Materials and Methods

### Plant Material

Analyses were performed on a subset of 112 industrial chicory lines originating from a collection described earlier (Dauchot et al., 2014). The 112 lines were created by selfing of 112 individual plants selected from a collection of 600 individuals randomly sampled out of 18 varieties at the origin of the selection of modern industrial chicory cultivars. The 112 individuals were selected using the Mstrat sampling strategy, based on 15 SSRs markers (Gouesnard et al., 2001) to gather maximum diversity of the original collection and minimize the structure of the sampled population.

The 112 lines were sown on May 2, 2011 in Warcoing (7740, Belgium) and 6 to 24 roots per line were harvested between Oct, 2 and Oct 9, 2011. These 112 lines were used for carbohydrate analysis, while the gDNA of the corresponding parents were used for FEH genotyping and qPCR analysis.

### Root Sampling and Carbohydrates Phenotyping

Due to the amount of material needed for carbohydrate analysis and the destructive nature of the procedure, analyses were performed on two pools of root tissues obtained after rasping several roots from the same line. This provided an estimate of the phenotype of the 112 parental plants at two distinct time points, at harvest and after harvest, respectively. The roots were phenotyped for five carbohydrate characteristics: the inulin degree of polymerization (DPin), inulin content (IN), sucrose content (SUCn), free fructose content (FFn), and free glucose content (FGn). The IN, SUCn, FFn, and FGn were expressed as a percentage of the total carbohydrate mass. Analyses were performed according to Van Waes (Van Waes et al., 1998) as described earlier (Dauchot et al., 2014).

### Leaf Sampling and DNA Extraction

Genomic DNA was extracted from the parent plants of the 112 lines. Upon sampling, fresh chicory leaves were stored in paper envelopes at -80°C until further processing. For gDNA extraction, frozen leaves were ground using a MM400 mixer mill (Retsch, Düsseldorf, Germany) for 45 s at 30 Hz in 35 ml stainless steel screw-top grinding jars (Retsch, # 01.462.0214) pre-cooled in liquid nitrogen. Leaf powder was immediately returned to -80°C until extraction. Hundred milligram of frozen leaf powder was used as starting material for genomic DNA extraction using NucleoSpin PlantII extraction kit according to manufacturer’s instructions (Macherey-Nagel, Duren, Germany). First step of the protocol uses a buffer containing RNAse. Following extraction, gDNA was evaluated for integrity by agarose gel electrophoresis and for purity by spectophotometric analysis at 260 and 280 nm on a Thermoscientific Multiskan GO device. 260/280 ratio quality threshold was set to 1.8 and could not exceed 2.1. Further validation of the quality and/or presence of PCR inhibitors was evaluated by performing a classical amplification followed, in case of positive amplification, by the evaluation of qPCR efficiency of DNA pools with qPCR primer pairs.

### PCR and qPCR Primer Design

Quantitative PCR primers were picked manually applying selection criteria as described earlier (D’haene et al., 2010). Melting temperature was calculated using the ABi online tool available at http://www6.appliedbiosystems.com/support/techtools/calc/ with a salt concentration of 43 mM and a primer concentration of 0.1 μM. Primers used for qPCR analyses are listed in **Table 1**. All primers were synthesized by Integrated DNA technologies (Leuven, Belgium) and were purified by standard desalting.

**Table 1:** Quantitative PCR (qPCR) amplification primers, Tm, and calculated efficiencies.

Target	PCR/qPCR	GenBank	Reference	Primer sequence	Temperature (°C)	Efficiency	Calibration curve	*R*^2^	Amplicons
Actin	qPCR	EF528575	Maroufi et al. (2010)	F: CCAAATCCAGCTCATCAGTCGR: TCTTTCGGCTCCGATGGTGAT	60	P1: 107.94 ± 3.48P2: Not tested P3: Not tested	-3.15 log Q0 + 24.43	0.999	gDNA: 74 bp cDNA: 74 bp
β-tubulin	qPCR	AF101419	Maroufi et al. (2010)	F: GCACGGCATTGATGTGACCR: GAACAAACCTCCCGCCACT	60	P1: 102.57 ± 4.37P2: 105.35 ± 2.14P3: 103.50 ± 3.27	-3.26 log Q0 + 25.60-3.20 log Q0 + 22.79-3.24 log Q0 + 25.41	0.9981.0000.999	gDNA: 101 bpcDNA: 101 bp
1-SST	qPCR	U81520	This paper	F: ATCTCCCATTCGCCATGGTR: TGCCAGTGTAGAGCATGATGATCT	60	P1: 104.24 ± 4.40P2: 96.22 ± 3.43P3: 99.90 ± 2.63	-3.22 log Q0 + 25.67-3.42 log Q0 + 25.61-3.32 log Q0 + 25.52	0.9980.9990.999	gDNA: 108 bpcDNA: 108 bp
1-FFT	qPCR	U84398	This paper	F: GAGCTTCCCGTAGCCTTGACR: CATTTCCGGTGTACAATGCA	60	P1: 103.88 ± 4.72P2: 98.83 ± 2.82P3: 100.55 ± 3.21	-3.23 log Q0 + 25.64-3.35 log Q0 + 25.49-3.31 log Q0 + 25.57	0.9970.9990.999	gDNA: 112 bpcDNA: 112 bp
1-FEH I	qPCR	AJ242538	This paper	F: TTTCTTCTCGAACCAGCTCTCAGR: CGTCTTGACCGGTATATAGAATTATGG	60	P1: 102.05 ± 2.18P2: 99.12 ± 2.65P3: 99.96 ± 2.61	-3.27 log Q0 + 22.73-3.34 log Q0 + 22.30-3.32 log Q0 + 22.63	0.9990.9990.999	gDNA: 118 bpcDNA: 118 bp
1-FEH IIa	qPCR	JQ585639	This paper	F: TCACACTTTGACCCCTTGGCTAR: GGGTAAATCCGACTTGTGATACATGTT	60	P1: 102.16 ± 3.38P2: 99.44 ± 2.32P3: 98.04 ± 3.77	-3.27 log Q0 + 23.64-3.34 log Q0 + 23.23-3.37 log Q0 + 23.47	0.9990.9990.998	gDNA: 103 bpcDNA: none
1-FEH IIb	qPCR	JQ585640	This paper	F: TCTTTACACTTTTGACCCCTTGTTTGR: GGGTAAATCCGACTTGTGATACATGTC	60	P1: 99.79 ± 4.59P2: 97.51 ± 3.06P3: 98.94 ± 4.3	-3.33 log Q0 + 24.70-3.38 log Q0 + 24.30-3.35 log Q0 + 24.56	0.9970.9990.998	gDNA: 105 bpcDNA: none

*According to plate design constraints, the 36 samples of this study were analyzed in three sets (P1, P2, and P3). For each set, gDNAs were pooled and used to estimate PCR efficiencies of five target genes and two normalization genes [Actin (ACT) and oxβ-tubulin]. P1 was used for first tests. Based on first test (P1), oxβ-tubulin was preferred as normalization gene*.

Prior to qPCR analysis, all gDNA were tested for amplifiable RNA/cDNA contaminants with a primer pair targeting a genomic region including an intron. All samples were checked for single band amplification profiles. The primer pair used for this test targets 1-FEH I (AJ2425378) and should amplify a 891 bp genomic fragment and only 395 bp on cDNA. Primer sequences were F- GCACTTTTCTAGTAAAACGGG and R- TCCGGTTATTGCTAAGCCAG. Amplifications were typically performed for 35 cycles with a Tm set to 55.C.

### qPCR Reaction Mixture and Cycling Conditions

Quantitative PCR reactions were performed in a 20 μl volume and were typically composed of 10 μl 2x GoTaq qPCR Master Mix (Promega, #A6001), 0.2 μl CRX internal reference (Promega, #A6001), 0.05 μl of 100 μM of each primer, gDNA and nuclease-free water to reach 20 μl. Reactions were all performed in ABi StepOnePlus instrument (96 wells) with ΔΔCT quantification method, SYBR green amplicon detection and Standard amplification conditions consisting of an initial denaturation step at 95.C for 10 min followed by 40 cycles at 95.C for 15 s and 60.C for 1min. Amplification stepwas followed by a melting curve analysis between 60 and 95.C.

### qPCR Efficiency

According to plate design constraints, gDNA were analyzed as three distinct DNA sets including intra- and inter-plate normalization reference samples. PCR efficiencies were calculated for each sample set treating them as three distinct pools of 16 to 21 individual gDNA whose concentration was normalized according to spectrophotometric quantification results. For each primer pair, efficiencies were determined based on 6 gDNA concentrations of 32, 8, 2, 0.5, 0.25, and 0.125 ng/μl. Serial dilution were performed using 5 ng/μl baker yeast t-RNA carrier solution (Ambion AM7119). For each target amplicon, two “no template control” (NTC) were also included: NTC1 used nuclease-free water as template, while NTC2 used t-RNA carrier solution as template. All tests were performed on the same qPCR plate in duplicate. Each 20 μl reaction contained 10 μl of 2x GoTaq qPCR Master Mix (Promega, #A6001), 0.2 μl of CRX internal dye (Promega, #A6001), 0.05 μl of each primer at initial concentration of 100 μM, 4.4 μl of nuclease-free water and 5.3 μl of the diluted gDNA. qPCR were performed on ABi StepOnePlusTM Real-Time PCR Systems with standard 2 h runs, comparative Ct quantification (ΔΔCt) and SYBR^®^ green detection parameters. PCR efficiencies were calculated with pyQPCR (available for download at http://pyqpcr.sourceforge.net/?static2/download), an interface for the qBase algorithm (Hellemans et al., 2007). PCR efficiency was calculated with the formula E = [10(–1/slope)–1]^∗^100. The slope of the linear regression was obtained when plotting CT value vs log of the theoretical copy number. Primer pairs were considered valid when calculated efficiency was between 90 and 110% with 100% as an optimum. Standard error was calculated with pyQPCR.

### Quantitative PCR

Normalized relative copy number was estimated on 36 samples. Due to plate design constraints, these samples were analyzed and treated as three distinct sample sets. To allow integration of the results, an identical normalization sample (X129) was used in the three pools. Three samples were also present across plates to evaluate the validity of the normalization and the inter-run reproducibility.

The three sample sets were extracted with the same protocol and followed the same validation pipeline. All samples were normalized to the same concentration (8 ng/μl) based on the results obtained during efficiency estimates in order to reach an average CT of 20. Efficiencies were evaluated individually for each target and each sample set to account for possible variability of DNA purity. Once prepared, a single gDNA dilution was performed for each sample and was used for all tests. Samples were not re-extracted or re-diluted between analyses to improve reproducibility.

Each plate included up to 22 test samples, one normalization sample common to all plates and two no template controls (NTC1 and NTC2). On a single plate, all tests were performed in duplicate. A 96 wells plate allowed the analysis of up to two target genes.

Each 20 μl qPCR reaction contained 10 μl of 2x GoTaq qPCR Master Mix (Promega, #A6001), 0.2 μl of CRX internal dye (Promega, #A6001), 0.05 μl of each primer at initial concentration of 100 μM, 4.7 μl of nuclease-free water and 5 μl of 8 ng/μl gDNA (40 ng). Amplification cycles were terminated by a melting curve analysis.

### Cloning and Sequencing of 1-FEH I and 1-FEH IIb Genomic Regions

For cloning, gDNA amplifications were all performed on two lines highly contrasted for their susceptibility to post-harvest inulin depolymerization, namely X191 (considered as depolymerization resistant) and X200 (considered as depolymerization-sensitive) lines.

### 1-FEH I

Partial genomic region of 1-FEH I was amplified with GoTaq Long Range PCR master mix (Promega) according to manufacturer’s instruction with a Tm of 54.3°C, 35 cycles, elongation time of 13 min in 10 μl volume with 100 ng of gDNA in an Eppendorf Master Cycler Pro S. Due to the high sequence homology between members of the GH32 family, the genomic region was amplified with a single primer pair using long range polymerases to avoid posterior assembly of chimeric sequences that might result from the amplification of multiple partial sequences.

The two primers 1-FEHI-96F CATTTGGGTTCTCTCTCTTTGC and 1-FEHI-1800-R CTAGGTTATAGTTACTAGAACATTATATG target the 96–1800 bp region of the 1-FEH I cDNA (AJ242538) which covers 98% (1672/1707 bp) of the corresponding coding CDS (61–1767). These primers amplified a single gDNA fragment of 10,575 bp (KM494975).

### 1-FEH IIb

Amplifications were performed with GoTaq Long Range PCR master mix (Promega) according to manufacturer’s instruction with a Tm of 50°C, 35 cycles, elongation time of 7 min in 10 μl volumes with 100 ng of gDNA with primers 1FEH2a-28F CTTTTTCTCCATATGTTGTCG and 1FEH2a-5744R CAAGGAATACAGCAACAAAGAATG.

For both 1-FEH I and 1-FEH IIb, inserts were gel-purified with Nucleospin Gel extraction kit (Macherey Nagel), inserts were cloned in PCR4.1 Topo vector (Invitrogen) and electroporated in TOP10 E. coli (Invitrogen). Sanger sequencing of the inserts was performed at Macrogen (The Netherlands).

### 1-FEH IIa and 1-FEH IIb Genotyping

The parents of each 112 lines used in this study were first genotyped with the primer pair specifically targeting a 47 bp duplication in the 3′UTR region of 1-FEH IIa as described previously (Dauchot et al., 2014).

They were then genotyped for the loss of the mini-exon 2. Loss of mini-exon 2 was monitored with primers F2ab-638F-AGAAACTACAAACTCATAAATGAATATGC and F2ab-865R-CTTTGAAATRTCTAGCCGCCGTAAC using GoTaq Long PCR master Mix (Promega # M4021) according to manufacturer’s instruction for 35x cycles at 54.3°C. This primer pair amplified a region located in the promoter of 1-FEH IIb and presented size polymorphism between the two 1-FEH IIb alleles (observed 247/302 bp; X200 – expected 250 bp, X191 – expected 305). Due to the high homology between 1-FEH IIa and 1-FEH IIb promoters, these primers also amplified 1-FEH IIa but with a distinct amplification size (observed 225/229 bp). The amplification size was in agreement with 1-FEH IIa genomic reference sequence (AY323935 – expected 228 bp).

### Data Analysis

Generation of NRQ values and standard error was performed using pyQPCR software as an interface to treat Abi StepOnePlus raw data with the qBase algorithm (Hellemans et al., 2007). ΔΔCT relative quantification was performed using β-tubulin as normalization gene.

### Statistical Tests

The association between the carbohydrate characteristics and the FEH genotype with the different NRQ values was analyzed, respectively, by ANOVA or linear regression, using the aov function of R version 3.0.0 (R Development Core Team, 2013). Model comparison was realized with the anova functions of R version 3.0.0 (R Development Core Team, 2013).

## Results

### Normalization Genes for qPCR in Chicory

This paper is, to our knowledge, the first one describing CNV analysis by qPCR in chicory. As a result, no normalization genes have been reported for this purpose so far. A paper reporting the validation of reference genes for real-time gene expression analysis (RT-PCR) in chicory evaluated the expression stability of NADHD, ACT, TUB, GAPDH, H3, EF, and rRNA and concluded that ACT, EF, rRNA, H3, and TUB could be used for normalization (Maroufi et al., 2010). Out of these genes, we tested ACT and TUB as normalization genes for CNV analysis.

Our results indicate that, based on a preliminary analysis performed on 20 individuals (Pool 1, P1), for our chicory sampling population, estimates of relative copy number of 1-FFT gene using ACT as normalization gene resulted in a higher variability and standard error deviation than when normalized against β-tubulin. In the initial test, efficiency of β-tubulin was also closer to the 100% optimum (102.57 ± 4.37) as compared to ACT (107.94 ± 3.48). β-tubulin was therefore used as a quantification reference. The apparent stability of 1-FFT copy number makes it a good candidate as an additional reference gene for copy number analysis in chicory.

### Specificity of qPCR Primer Pairs

For all tests, terminal melting curve analysis never identified any multiple melting temperatures. Specificity of ACT and tubulin primers was validated previously (Maroufi et al., 2010). The ACT qPCR primer pair was valid for use on cDNA as well as on gDNA (same amplification size). Tubulin qPCR primer pair was also located inside a single exon according to gDNA amplification size and personal unpublished gDNA sequences. 1-SST, 1-FFT, and 1-FEH I specific primer pairs were all located within a single exon. 1-FEH IIa and 1-FEH IIb are two paralog sequences presenting more than 94% homology of their coding sequence. Based on four partial gDNA sequences published recently (JQ585641, JQ585640, JQ585639, and JQ585638), highly specific primer pairs could be designed to amplify a region covering part of the last intron and part of the last exon. This design ensured a high specificity for qPCR use. All primer pairs and accession numbers of their respective target sequences are listed in **Table 1**.

### qPCR Efficiencies

Efficiencies of qPCR primer pairs were calculated as explained in the material and methods section. Results are summarized in **Table 1**. Efficiencies of all primer pairs included in this study were between 96.2 and 105.4%. Efficiencies were estimated for each gDNA set independently to account for variability between the investigated gDNA sets.

### Association of GH32 NRQ Data With Post-Harvest Inulin Depolymerization

We investigated the relative copy number of five members of the GH32 family by qPCR on the genomic DNA of the parents of 36 lines used for the phenotyping.

**FIGURE 1:**
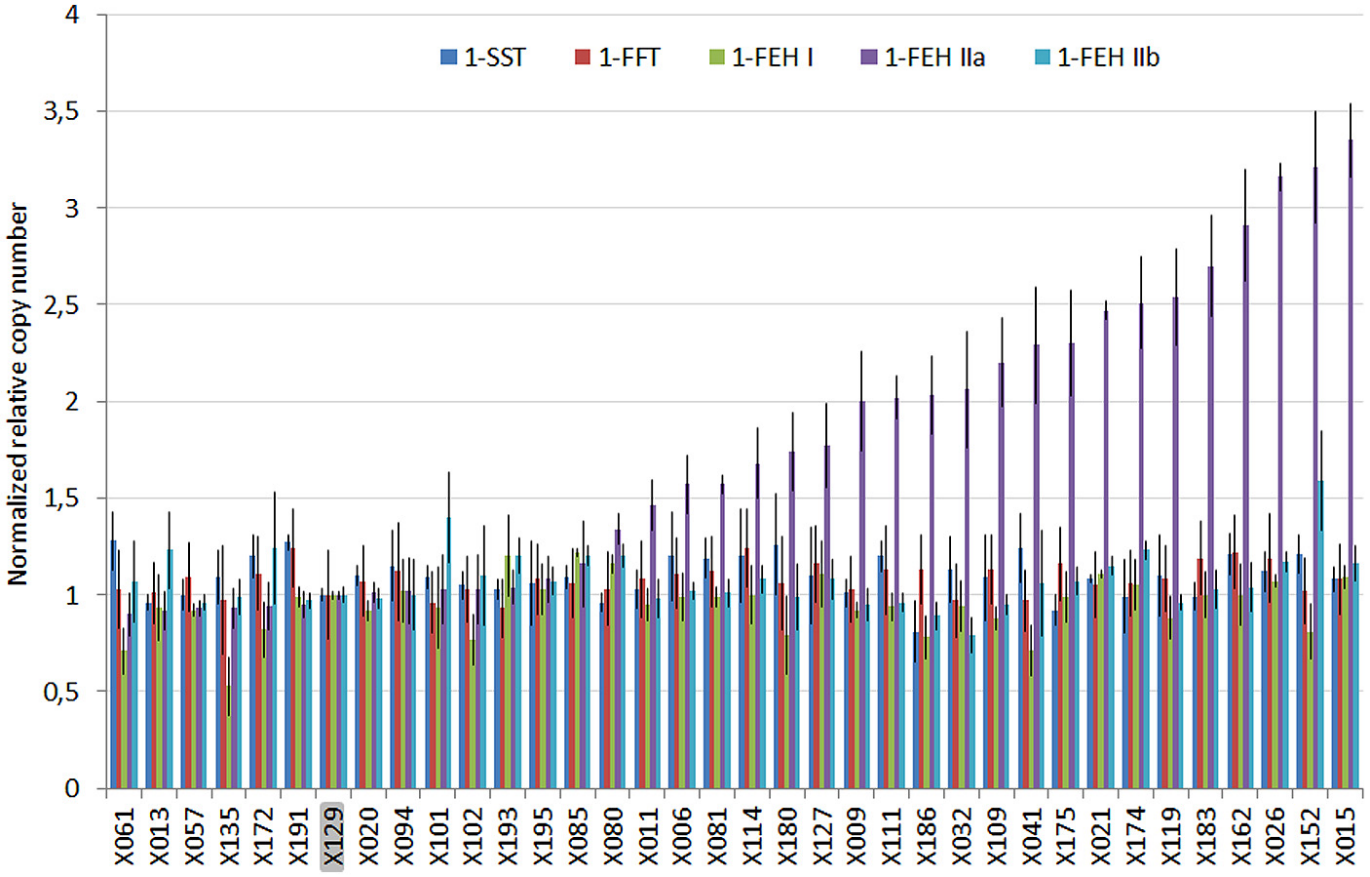
**Plot of the normalized relative quantities (NRQ) and standard error estimated for five members of the GH32 multigenic family by qPCR on the genomic DNA of 36 chicory lines exhibiting different susceptibility to post-harvest inulin depolymerization.** X129 was used to normalize the results. Significant differences of relative copy number were only detected for 1-FEH IIa.

The NRQ values for 1-SST, 1-FFT, 1-FEH I, and 1-FEHIIb showed a small variability, ranging from 0.81, 0.93, 0.53, 0.79 to 1.28, 1.24, 1.22, and 1.59, respectively, while 1-FEH IIa showed a wider range of NRQ values ranging from 0.90 up to 3.35 (**Figure 1**).

**FIGURE 2:**
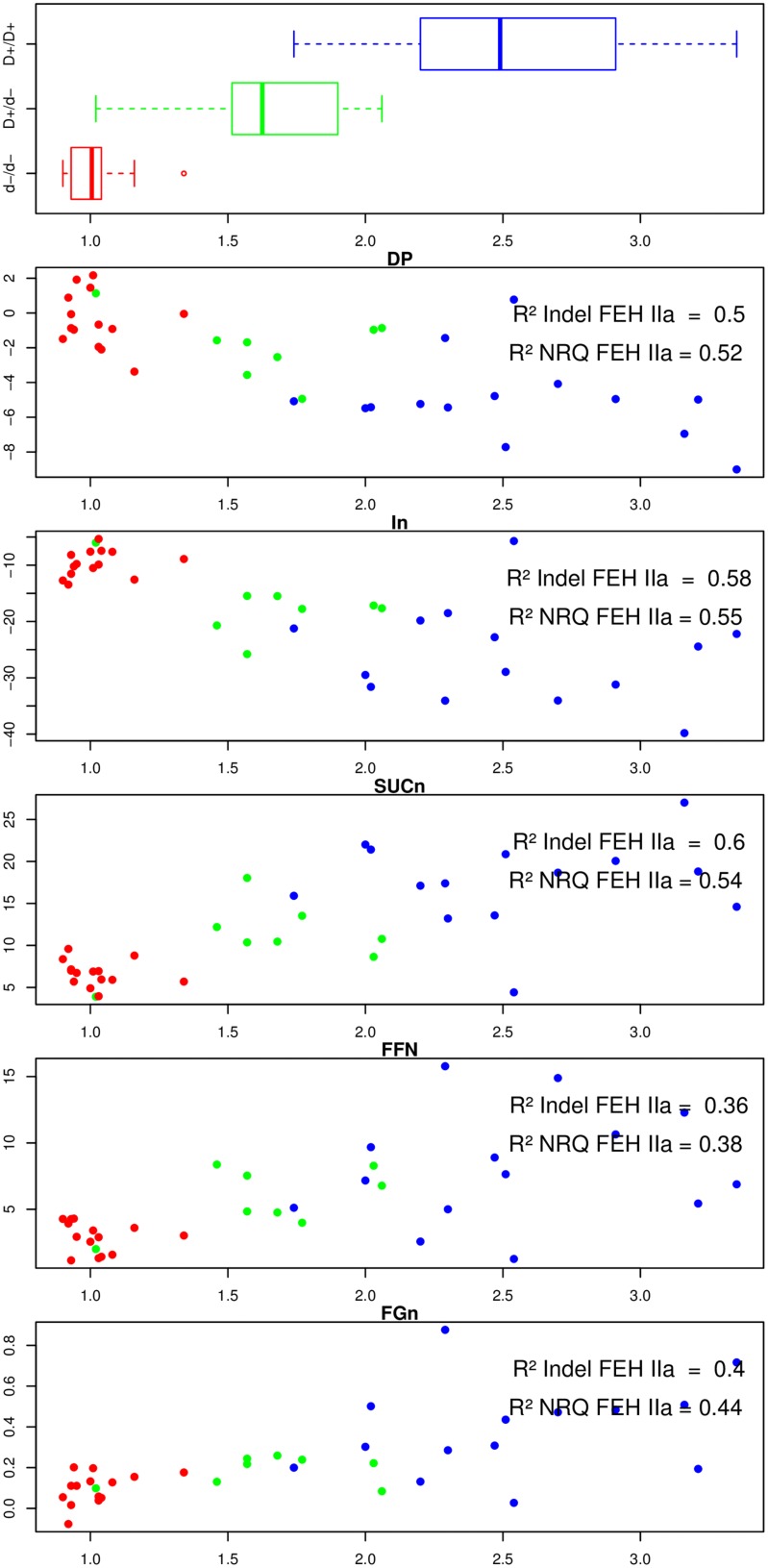
**For 36 lines, boxplot of the genotype of the duplication located in 3′UTR region of 1-FEH IIa, plot of the difference of the inulin degree of polymerization (DPin), inulin content (IN), sucrose content (SUCn), free fructose content (FFn), and free glucose content (FGn) before and after exposure to post-harvest cold temperatures (*Y* axis) according to the FEH IIa NRQ value (*X* axis)**. Each data point is colored according to the nature of the FEH genotype (3′ duplication) previously associated with contrasted susceptibilities to post-harvest inulin depolymerization. Blue: homozygous for the FEH IIa allele presenting no duplication in the 3′ UTR, red: homozygous for the FEH IIa allele presenting the 47 bp duplication in its 3′ UTR, green: heterozygote.

We performed statistical tests to evaluate the correlation between NRQ values and phenotypic data recorded at harvest and after harvest. These results are presented in **Figure 2**

For each plot, we also included a three color code, referring to the genotype of 1-FEH IIa. We previously demonstrated a strong correlation between the presence/absence of a 47 bp duplication in the 3′UTR of 1-FEH IIa and the susceptibility to post-harvest inulin depolymerization. A graphical representation to this 47 bp duplication can be found in the supplementary material (ESM_5) of our previous paper (Dauchot et al., 2014). **Figure 2** also presents the R^2^ values obtained for the presence/absence of the 47 bp long duplication in the 3′UTR of 1-FEH IIa (Model 1) and for the loss of mini-exon 2 in 1-FEH IIb (Model 2), respectively.

Among the 36 parents analyzed by qPCR, 15 plants were genotyped as being homozygous for the absence of duplication in the 3′UTR (red: d–/d– considered as more resistant to depolymerization), 14 plants were homozygous for the presence of duplication (blue:D+/D+, considered as depolymerization prone), and 8 heterozygous plants (green: D+/d- presenting an intermediate phenotype).

For these three categories, the 1-FEH IIa NRQ values gave averages of 1.02, 1.64, and 2.5, respectively, (**Figure 2**, upper box). The variability in 1-FEH2a relative copy number was very low for the d-/d-, higher for the D+/d- and highest for D/D. The genotype for the 1-FEH IIa 3′ duplication was significantly correlated and explained and important proportion of variation of the 1-FEH IIa normalized relative copy number (*p*Val = 3.2^∗^ 10^-12^ *R*^2^ = 0.78). From these results, we conclude that the presence of a perfect 47 bp tandem duplication in the 3′UTR of 1-FEH IIa is correlated with the presence of multiple copies of 1-FEH IIa. Low 1-FEH IIa copy number is, on the other hand, correlated to the absence of the 47 bp tandem duplication.

We could not detect any other significant association between the genotype of the 1-FEH IIa 3′ duplication and the NRQ values of the four other GH32.

We then investigated a putative quantitative effect of 1-FEH IIa copy number on the carbohydrate-related phenotypic data recorded at harvest and after harvest (**Figure 2**).

At harvest, no significant association (*p* > 0.01) could be detected between all carbohydrate phenotypic data and any of the NRQ data of the five GH32. As previously reported, at harvest, the 1-FEH IIa 3′ duplication did not correlate with any difference of carbohydrate content or properties (Dauchot et al., 2014).

After harvest, both the 1-FEH IIa 3′ duplication marker and 1-FEH IIa relative copy number, considered at a global level, were strongly and significantly correlated with the variation during the storage of five carbohydrate-related parameters (*p*-value raged from 0.0002 to 10^-8^). Those two polymorphisms explained an important proportion of the variability of the different carbohydrate-related parameters with *R*^2^ values ranging from 0.36 to 0.60 (**Figure 2**). The proportion of variance explained by the 1-FEH IIa 3′ duplication (Model 1) and 1-FEH IIa NRQ (Model 2) were not significantly different (*p* > 0.01; Supplementary Table S1). The results observed for 1-FEH IIa NRQ are more than likely a reflection of structuration associated with the 1-FEH IIa 3′ duplication, rather than a quantitative effect of 1-FEH IIa copy number: actually, when the three classes of genotypes (1-FEH IIa d-/d-, D+/d- and D+/D+) were analyzed independently, no significant association with any carbohydrate-related phenotype was detected within any class. These results suggest that copy number, by itself, has no direct quantitative effect on the susceptibility to post-harvest inulin depolymerization.

### Identification of a Loss-of-Function Allele of 1-FEH IIb

To further characterize the molecular basis of the difference of susceptibility to post-harvest inulin depolymerization, we set out to clone and sequence full length genomic sequences of the 1-FEH I, 1-FEH IIa, and 1-FEH IIb genes in depolymerization-prone and less susceptible chicory lines. Characterization of the multiple copies of 1-FEH IIa was of major interest.

Despite several trials with numerous combinations of primers, cycling conditions and the use of long range DNA polymerases, we were unable to re-amplify the previously published 1-FEH IIa genomic sequence (AY323935) in any of our biological samples.

However, using a primer pair initially designed to amplify a large genomic region of 1-FEH IIa (AY323935), we amplified a genomic region covering 100% of the cds of 1-FEH IIb in less susceptible (6154 bp – KM494977 ) and depolymerization-prone lines (7235 bp – KM494976). Comparison with 1-FEH IIb cDNA sequences (AJ295034) confirmed that these genomic sequences, amplified as single fragments, did correspond to 1-FEH IIb. Previously published partial genomic sequences of 1-FEH IIb (JQ585641 and JQ585640) align with these two larger genomic sequences.

**FIGURE 3:**
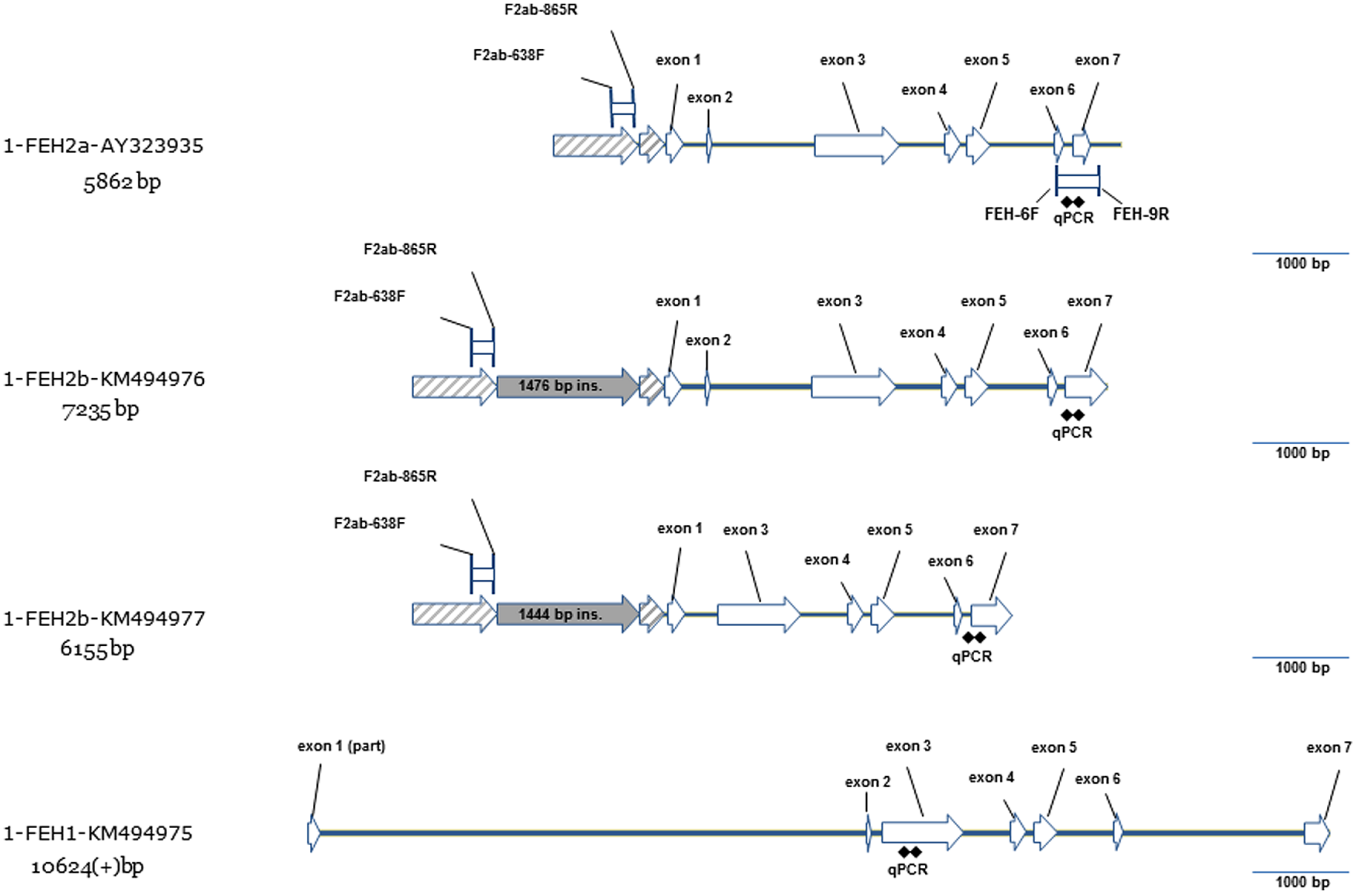
**Gene model for three new genomic sequences of 1-FEH I (KM494975) and 1-FEH IIb (KM494976, KM494977) compared to the previously published gene model of 1-FEH IIa (AY323935).** 1-FEH IIb (KM494976) has an intron-exon structure similar to 1-FEH IIa with six introns and seven exons. Promoter region of 1-FEH IIa and 1-FEH IIb share two collinear blocs splitted in 1-FEH IIb by a large insertion of 1.5 kb. Compared to KM494976, the sequence KM494977 presents a 1.028 bp deletion which covers part of introns 1 and 2 and the entire mini-exon 2. Compared to the 1-FEH II group, partial genomic sequence of 1-FEH I is much larger, presents a large first intron and a very short second intron. This structure is typical of cell wall invertases. In 1-FEH II, the second intron is larger than the first one. This structure is more similar to what is observed in vacuolar invertases.

According to the published genomic sequence of 1-FEH IIa (AY323935), 1-FEH IIb genomic sequence shares a number of similarities, including the same intron/exon structure (**Figure 3**). The only noticeable difference is located in the region upstream of the initiation codon: 1-FEH IIa and 1-FEH IIb present two collinear blocks which are split by a large insertion of approximately 1.5 kb in 1-FEH IIb. When comparing the two 1-FEH IIb alleles (KM494976 and KM494977) isolated from depolymerization prone and resistant lines, respectively, we noticed the presence of a large deletion (1018 bp) in KM494977. This deletion includes the 9 bp-long mini-exon 2 and parts of introns 1 and 2. This result is of particular interest since mini-exon 2 contains one of the three conserved active site residues. The loss of this residue must result in the inactivation of the 1-FEH IIb allele (X191 – KM494977).

We were also able to amplify as a single fragment, a 10,575 bp long region of 1-FEH I in a depolymerization-resistant line (X191 – KM494975) but not in the tested susceptible line (X200). This fragment covers part of the entire cds at the exception of two short regions at the beginning of exon 1 and at the end of exon 7 (**Figure 3**).

Our cloning strategy, using long range DNA polymerase and unique primer pairs, allowed the amplification and sequencing of new 1-FEH I and 1-FEH IIb gDNA sequences. This strategy ensures that sequences are not chimeric: this point is of particular importance considering the high homology (96%) of the cds of 1-FEH IIa and 1-FEH IIb. The genomic sequence of 1-FEH IIa (AY323935), published much earlier (Michiels et al., 2004), was amplified in tree fragments with stringent annealing conditions but poorly specific primer pairs. The overlap between the tree fragments was also very limited. The risk that AY323935 is a chimeric sequence, or a rare recombinant allele, is then present and supported by the impossibility to re-amplify this full length sequence as a single fragment in our biological material. Moreover, using primers designed to amplify 1-FEH IIa genomic region (AY323935), we amplified the full length genomic sequence of 1-FEH IIb instead. In the same paper (Michiels et al., 2004), the authors mention that the intron/exon structure of 1-FEH IIa is closer to vacuolar invertase than to cell wall invertase, due to the presence of a large second intron which is typical of vacuolar invertase, while in cell wall invertase, the first intron is larger than the second one. The intron/exon structure of 1-FEH IIb supports its relationship with vacuolar invertase. The gene structure of 1-FEH I, on the contrary, highlights the presence of a very large first intron (5693 bp) and a short second intron (90 bp), which is typical of cell wall invertases (**Figure 3**). This result further discriminates chicory 1-FEH I from the 1-FEH II a/b group.

### Impact of the Loss of Mini-Exon 2 in FEH IIb

We genotyped 112 samples to evaluate the impact of the loss of mini-exon 2 in 1-FEH IIb on the susceptibility to post-harvest inulin depolymerization and to compare these results to those obtained on the same samples with the presence/absence of the 47 bp duplication in the 3′UTR of 1-FEH IIa. The results indicate that these two loci are closely linked. Indeed, except for eight individuals, the absence of 3′ duplication in 1-FEH IIa was associated with the loss of mini-exon 2 in 1-FEH IIb (Supplementary Table S2), which results in a very high linkage disequilibrium between the two loci (*r*^2^ = 0.89).

The two loci were both significantly associated with the different carbohydrate characteristics with *R*^2^ values ranging from 0.26 to 0.44 (Supplementary Table S3). The proportion of the variance explained by the two polymorphisms was not significantly different for the five carbohydrates parameters (*p* > 0.01). Raw genotyping results are presented as supplementary material (Supplementary Table S4).

These results indicate that loss of mini-exon 2 in 1-FEH IIb, which results in the loss of one of the three residues of the active site, had a strong impact on the susceptibility to post-harvest inulin depolymerization. This supports the implication of 1-FEH IIb in the depolymerization of inulin induced by exposure to end-season cold temperatures.

## Discussion

Ultra-high throughput sequencing drastically speeds-up the discovery of SNPs and provides tremendous amounts of new genomic data. All these sequences coupled with the development of CGH arrays highlight the abundance of genomic rearrangements among them the underestimated CNV. Compared to SNP, CNV in the human genome could be at least 1000 up to 10,000-fold more frequent (Lupski, 2007). It is only recently that CNV in animal and plant genomes have been identified as a significant source of phenotypic variation: CNV might represent a new milestone in the understanding of the genetic basis of quantitative traits variability.

However, while we were able to clearly identify CNVs in the 1-FEH IIa gene, the results presented here provide an example where CNV of functional candidate genes had no quantitative effect on the phenotype.

From a mechanistic point of view, three models are proposed to explain the emergence of CNV in genomes: the Non Allelic Homologous Recombination (NAHR) mostly mediated by low-copy repeats (LCRs), the Non-Homologous End-Joining model (NHEJ) and the fork stalling and template switching (FoSTeS) model. CNV are further described as recurrent when rearrangements have common size and breakpoints, non-recurrent when CNV share a common region but where breakpoints are located at various positions, and non-recurrent with grouping, when CNV share a common breakpoint which might result from an underlying genomic architecture such as palindrome or cruciform structure (Gu et al., 2008; Stankiewicz and Lupski, 2010).

In our study, we identified variations of copy number only in the 1-FEH IIa allele presenting a 47 bp duplicated region in its 3′UTR (JQ585639). Since we never observed multiple copies of the “short” 1-FEH IIa allele (JQ585638), we hypothesize that this duplication might represent a genomic structure favoring the generation of multiple copies.

Regarding the absence of a quantitative effect of the CNV of 1-FEH IIa, we hypothesize that some of the 1-FEH IIa copies might be truncated or pseudogenized and result in non-functional copies. Being located at the 3′end of 1-FEH IIa gene, in case of incomplete copies, the primer pairs we used for CNV estimation cannot discriminate between 5′truncated and full-length functional copies, which prevents us from drawing conclusions on any potential additive effect of additional copies of the gene on the phenotype. To estimate the number of functional 1-FEH IIa copies, one should consider performing qRT-PCR on 1-FEH IIa with sequence-specific primers. However, the very high identity of 1-FEH IIa and 1-FEH IIb cds (96%) makes it very difficult to design 1-FEH IIa specific primer pairs meeting qRT-PCR requirements. In the past, despite extensive trials, we were only able to specifically amplify 1-FEH IIb transcripts for semi-quantitative RT-PCR. At that time, we used very stringent annealing conditions and large amplicons which are incompatible with modern qRT-PCR. Amplification of 1-FEH IIa alone was never achieved (Supplementary Figure S1).

Although CNV was not detected in 1-FEH IIb, the sequencing of 1-FEH IIb genomic region revealed the presence of an 1-FEH IIb allele missing the mini-exon 2. This particular allele was observed in chicories less susceptible to post-harvest inulin depolymerization. Genotyping of a larger set of 112 samples revealed that the absence of the duplication in the 3′UTR of 1-FEH IIa was correlated to the absence of multiple copies of 1-FEH IIa and to the loss of mini-exon 2 in 1-FEH IIb. This observation might result from the physical proximity of 1-FEH IIa and 1-FEH IIb, which were previously located on the LG4 of the Rubis 118 map at 28.8 and 30.6 cM, respectively, (Cadalen et al., 2010).

**FIGURE 4:**
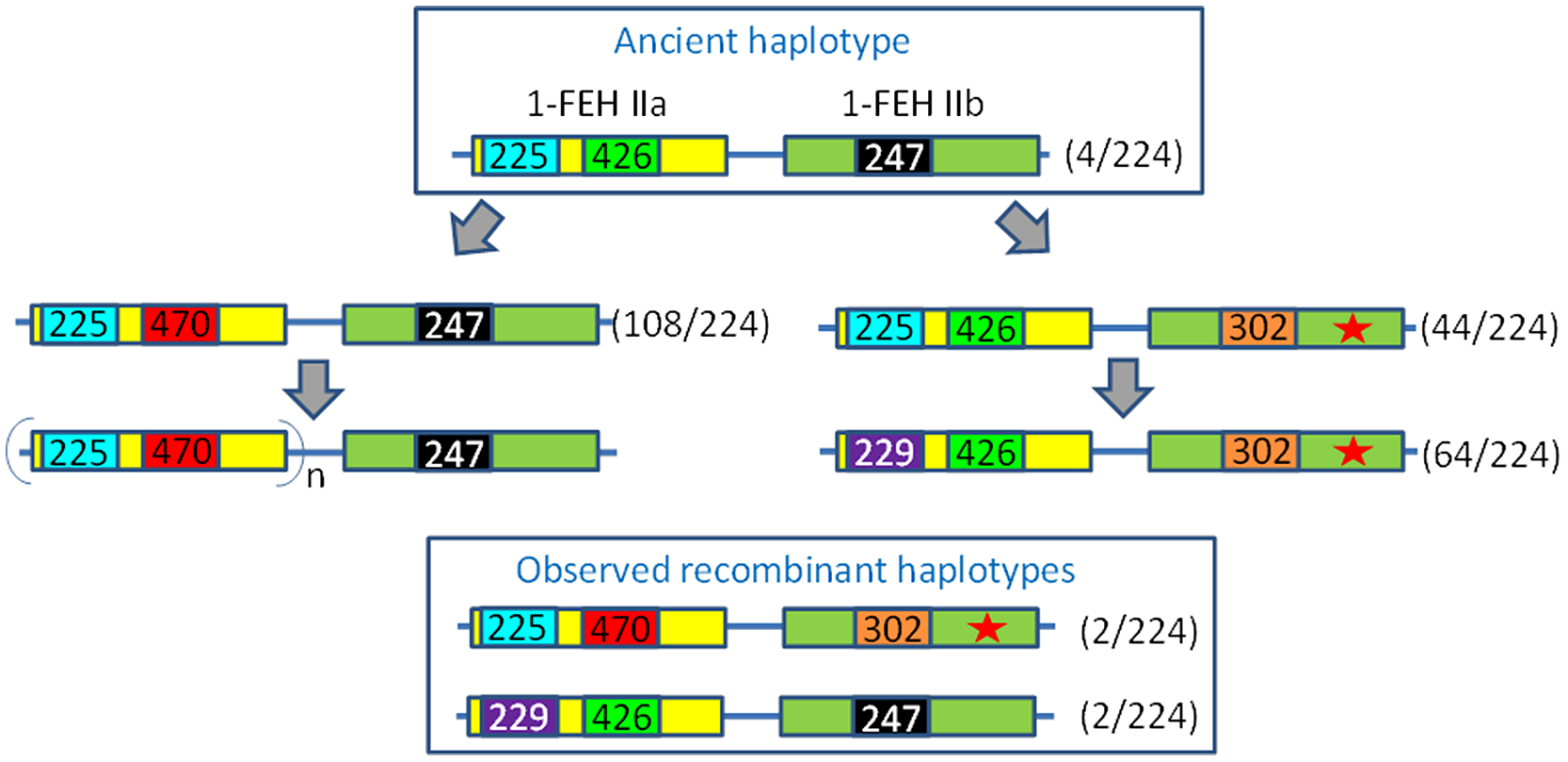
**Evolution of the 1-FEH IIa/IIb haplotypes.** Numbers reported in the figure refer to the size of PCR products such as scored on an ABi 3130Xl fragment analyzer. The haplotypes are defined based on a locus located in the promoter region of 1-FEH IIa (225/229), a locus in the 3′UTR of 1-FEH IIa (426/470) and one locus in the promoter region of 1-FEH IIb (247/302) which was shown to be associated with the loss of mini-exon 2 in the same sequence (Red star). The 225/426/247 haplotype is considered ancestral. The 470 allele originates from a tandem duplication of a 47 bp region in the 3′UTR of 1-FEH IIa. This duplication is perfect, which suggests it happened recently. Moreover, this duplication was not detected in other Asteracean 1-FEH II (see Supplementary Figure S1). This genomic structure might favor the mechanism of CNV since multiple copies of 1-FEH IIa were only observed with this haplotype. The 1-FEH IIb-302 allele was only observed in combination with the ancestral 1-FEH IIa-426 allele. The 1-FEH IIa-229 allele appeared after the loss of mini-exon 2. Four putative recombinant haplotypes were identified. Values under brackets indicate the number of haplotypes detected among 112 diploid individuals.

Based on these results, we identified several haplotypes. We propose an evolutionary scheme to explain the emergence of these haplotypes (**Figure 4**). Two evolutionary lines are proposed, starting from an ancient haplotype consisting of the “short” 1-FEH IIa allele (426 bp) and a functional 1-FEH IIb allele (identified by 247 bp locus). This ancestral haplotype evolved into a new haplotype harboring a 47 bp tandem duplication in the 3′end of 1-FEH IIa (470 bp locus). This haplotype likely evolved and acquired multiple copies of 1-FEH IIa. This 47 bp duplication is considered “recent,” as the tandem repeat is perfectly identical and such duplication was never observed in other Asteraceae (Supplementary Figure S2). The 1-FEH IIa “copy expansion” is not observed in the second haplotype group (red star in **Figure 4**) which underwent the loss of mini-exon 2 and an insertion in the promoter region (extending it from 247 to 302 bp). Later modification of the 1-FEH IIa promoter region (225 bp) which led to a sequence increase up to 229 bp was only observed in this haplotype group. This evolutionary scheme is parsimonious and also explains the existence of two recombinant haplotypes.

To fully understand the importance of the loss of the 9 bp mini-exon 2 in 1-FEH IIb, one should remember that all GH32 enzymes are composed of a C-terminal β-sheet and a N-terminal β-propeller. In 1-FEH IIa, 3D modeling positions the active site in the center of the β-propeller domain. The active site of 1-FEH IIa is a negatively charged pocket which contains three acidic residues D22 (from the NDPNG conserved sucrose binding box covering mini-exon 2), D147 (from FRDP), and E201 (from WECPD). In the reaction scheme proposed for 1-FEH IIa, E201 protonates the glycosidic oxygen (acid/base catalyst) while the carboxylate group of D22 is used for a nucleophilic attack to produce a covalent fructose enzyme intermediate. The intermediate is then hydrolyzed to release a free fructose, a (n-1) inulin chain and the free enzyme (Verhaest et al., 2005).

**FIGURE 5:**
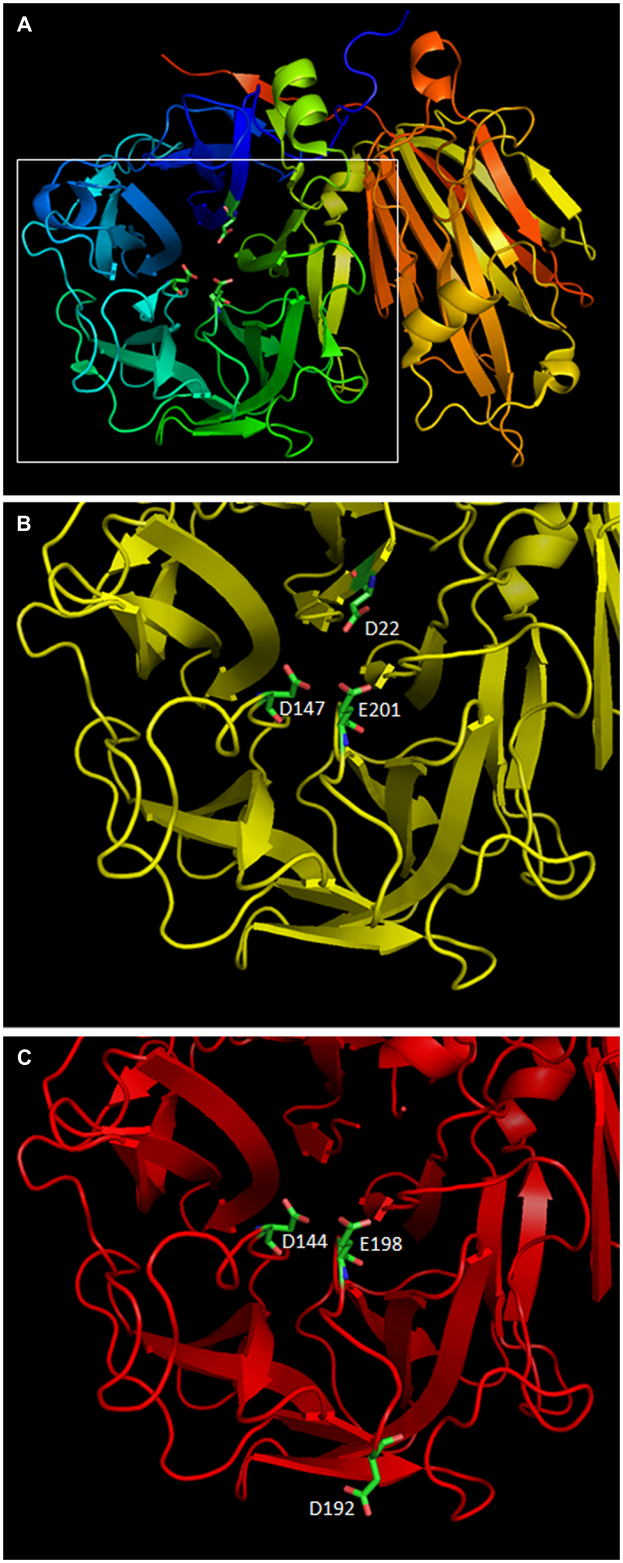
**Homology modeling of the two 1-FEH IIb alleles identified in this paper was performed using the 3D model of chicory 1-FEH IIa (1ST8) with SWISS-MODEL online tool.** PDB files were manipulated using PyMol. **(A)** 3D model of the wild type 1-FEH IIb allele (AIP90173). The active site is located inside the β-propeler structure which contains three active residues: D22, D147, and E201. **(B)** zoom on the active site of AIP90173 **(C)** model of the active site of the deleted 1-FEH IIb allele (AIP90174). The loss of the DPN triplet is highlighted by a gap in the 3D model. D144 and E198 have the same positions and orientations as the original D147 and E201 residues. A new D192 residue is observed on the outide of the protein. D192 is too far from the active site to compensate for the loss of D22. AIP90174 is, as a result, more than likely non functional.

The importance of the D residue located in the sucrose binding box was demonstrated as early as 1990 when the mutation of D23 in a yeast Invertase made it basically inactive (Reddy and Maley, 1990). Later, mutation of D23A in AtcwINV1 resulted into a 900-fold reduction of K_cat_ as compared to the wild type enzyme, confirming the crucial role of this residue (Le Roy et al., 2007). In this context, the loss of mini-exon 2 in 1-FEH IIb, which results into the loss of the DPN triplet inside the sucrose binding box, which itself contains the mandatory D residue, results, more than likely, into an inactive allele of 1-FEH IIb. To further compare both 1-FEH IIb alleles, homology modeling was performed with SWISS-MODEL starting from the available 1-FEH IIa 3D model (1ST8) published earlier (Verhaest et al., 2005). 3D models of both 1-FEH IIb alleles are highly similar (**Figures 5A–C**). The only noticeable differences are the loss of the DPN triplet which results into the loss of the D22 residue in the active site and the apparition of a new D192 residue in the deleted 1-FEH IIb allele. However, this new D residue is located too far from the active site to compensate for the loss of D22. No other mutations on this allele could reasonably restore its activity (Supplementary Figure S3).

The loss of mini-exon 2 is then of particular interest to explain the lower susceptibility to post-harvest inulin depolymerization. We confirmed experimentally that the loss of 9 bp of the coding sequence of 1-FEH IIb is not associated with a reading frame shift and that this truncated version can still be transcribed normally (Supplementary Figure S4).

An increase of the FEH enzymatic activity following exposure to end-season cold temperature has been reported (Van den Ende and Van Laere, 2002). However, at the protein level, the relative contribution of the three FEH enzymes to the total activity is almost impossible to determine. To tackle this question, the same authors published a second paper reporting the seasonal evolution of transcription level of several members of the GH32 multigenic family as analyzed by Northern blot. The results were, at that time, interpreted as illustrative of a higher specificity of cold-regulation of 1-FEH IIa, as compared to 1-FEH I (Van den Ende et al., 2002). However, results for 1-FEH IIb were not reported. Considering the high homology between the cds of 1-FEH IIa and 1-FEH IIb (96%), one could reasonably question the specificity of the Northern blot probes used for 1-FEH IIa and consider these Northern blots as illustrative of the expression level of the 1-FEH IIa/b rather than 1-FEH IIa alone. This was taken into account in a later publication where the authors mention that “mRNA blot analysis might also detect other isoforms” (Michiels et al., 2004). In this paper, promoter deletions of 1-FEH IIa and resulting modulation of transcription by cold was studied in detail, but no information regarding the regulation of 1-FEH IIb was mentioned. Personal unpublished results also highlighted the specific transcriptional up-regulation of 1-FEH IIb following exposure to end-season cold temperatures of chicories in the field (Supplementary Figure S1).

Even if our results do not rule out the specific over-expression of 1-FEH IIa in response to cold-induced inulin depolymerization, they suggest that multiple copies of 1-FEH IIa, as detected by qPCR, have no direct quantitative effect on the susceptibility to post-harvest inulin depolymerization. Questions regarding the non-functional nature of some of these 1-FEH IIa copies remain to be answered. The absence of statistical correlation might result from the presence of truncated or pseudogenized copies of 1-FEH IIa, interfering with the evaluation of a quantitative effect. On the other hand, our results demonstrate that the loss of a functional 1-FEH IIb allele has a direct and significant impact on post-harvest inulin depolymerization and related free sugars content. This underlines the contribution of 1-FEH IIb to inulin depolymerization after harvest. More generally, we suggest that CNVs should be evaluated with several probes located in the promoter and the 5′ and 3′ regions of the genes of interest to account for possible truncation, eventually coupled with qRT-PCR to provide an accurate estimate of functional copies.

Our results highlight the presence of a non-functional allele of 1-FEH IIb. In this context, association between expression levels and inulin depolymerization has to take into account the nature of the 1-FEH IIb allele. The same remark could apply to 1-FEH IIa if the presence of multiple incomplete copies was confirmed. The methodology and probes developed here can help answering these questions.

## Conclusion

Here we established for the first time the existence of CNV in the GH32 multigenic family. We also demonstrated that CNV, even of a clear candidate gene, is not necessarily responsible for phenotypic variation and needs careful examination of neighboring regions. The discovery of a new loss-of-function allele of 1-FEH IIb whose presence was clearly associated with lower propensity to post-harvest inulin depolymerization supports the implication of 1-FEH IIb in a specific cold stress response adaptation in chicory.

## Author Contributions

ND wrote most of the article and performed the qPCR, cloning, sequencing and FEH genotyping. PR performed the statistical analyses and selected the 112 lines. OM and his team performed DNA extractions and SSR genotyping to generate the data used for the selection of the 112 lines. They also performed all the carbohydrate-related phenotyping. CN and her team created the first sample set and managed all biological samples and fields experiments. XD supervised statistical analysis and contributed to the revision of the manuscript. PVC supervised the entire study and significantly contributed to the revision of the manuscript.

## Ethical Statement

The authors acknowledge that the experiments described in this paper comply with the current laws of the country in which they were performed.

## Conflict of Interest Statement

Olivier Maudoux and Christine Notté are members of Cosucra – Group Warcoing S.A. The authors declare that the research was conducted in the absence of any commercial or financial relationships that could be construed as a potential conflict of interest.

## ABBREVIATIONS

ACT: actin
CNV: copy number variation/variants
EF: elongation factor 1-alpha
FEH: fructan exohydrolase
GAPDH: glyceraldehyde 3-phosphate-dehydrogenase
GH32: glycosyde hydrolase 32
H3: histone H3
NADHD: nicotinamide adenine dinucleotide dehydrogenase
NRQ: normalized relative quantities
qPCR: quantitative PCR (performed on gDNA)
rRNA: 18S rRNA
TUB: beta-tubulin
