# Supplementary material for: Loss of function of 1-FEH IIb has more impact on post-harvest inulin degradation in Cichorium intybus than copy number variation of its close paralog 1-FEH IIa
Source: Front Plant Sci. 2015 Jun 23;6:455. doi: 10.3389/fpls.2015.00455 (PMC4477480; doi:10.3389/fpls.2015.00455)
Supplement: Supplementary file 1 [file Table_1.PDF]

Loss of function of 1-FEH IIb has more impact on post-harvest inulin degradation in *Cichorium intybus* than copy number variation of its close paralog 1-FEH IIa. Nicolas Dauchot[(*)] . Pierre Raulier . Olivier Maudoux . Christine Notté. Xavier Draye . Pierre Van Cutsem.
[(*)]Research Unit in Plant Biology, University of Namur, 61 rue de Bruxelles, 5000 Namur, Belgium e-mail: nicolas.dauchot@unamur.be
Frontiers in plant science

Supplementary table 1: Comparison of the variance explained by the presence/absence of a 47 bp in the 3'UTR of 1-FEH IIa and the normalized relative copy number of the same gene (as estimated by qPCR). Depending on the carbohydrate parameters (DP – degree of polymerization, IN – inulin content, Suc – free sucrose content , Fru – free fructose content, Glu – free glucose content), the two models explained from 36% up to 60% of the variance. 1-FEH IIa copy number did not increase the proportion of the variance explained by the indel model.

	DP.R²	DP.Pval	IN.R²	IN.Pval	Suc.R²	Suc.Pval	Fru.R²	Fru.Pval	Glu.R²	Glu.Pval
indel FEH IIa	0,5028	3,72E-06	0,5754	2,75E-07	0,5969	1,17E-07	0,3567	0,0002614	0,4011	8,03E-05
NRQ FEH IIa	0,5246	3,58E-07	0,5454	1,65E-07	0,5366	2,30E-07	0,377	4,07E-05	0,4425	5,77E-06
