# Supplementary material for: Loss of function of 1-FEH IIb has more impact on post-harvest inulin degradation in Cichorium intybus than copy number variation of its close paralog 1-FEH IIa
Source: Front Plant Sci. 2015 Jun 23;6:455. doi: 10.3389/fpls.2015.00455 (PMC4477480; doi:10.3389/fpls.2015.00455)
Supplement: Supplementary file 2 [file Table_2.PDF]

Loss of function of 1-FEH IIb has more impact on post-harvest inulin degradation in *Cichorium intybus* than copy number variation of its close paralog 1-FEH IIa. Nicolas Dauchot(*) . Pierre Raulier . Olivier Maudoux . Christine Notté. Xavier Draye . Pierre Van Cutsem. (*)Research Unit in Plant Biology, University of Namur, 61 rue de Bruxelles, 5000 Namur, Belgium e-mail: nicolas.dauchot@unamur.be
Frontiers in plant science

Supplementary table 2: Correlation table obtained for 112 individual for two genetic markers located in 1-FEH IIa and 1-FEH IIb. The two markers are highly correlated (r^2 = 0.89). "d-/d- 1-FEH IIa" stands for "homozygous for the absence of duplication" in the 3' UTR of 1-FEH IIa, "D+/D+ 1-FEH IIa" stands for "homozygous for the presence of duplication" in the 3'UTR of 1-FEH IIa and "d-/D+" corresponds to heterozygous genotypes. "D+/D+ 1-FEH IIb" stands for "homozygous for the loss of mini-exon 2 in 1-FEH IIb", "d-/d-" stands for homozygous for the normal 1-FEH IIb allele" and "d-/D+" corresponds to heterozygous genotypes.

		1-FEH IIa		
		d-/d-	D+/d-	D+/D+
1-FEH IIb	d-/d-	0	6	36
	D+/d-	0	31	1
	D+/D+	37	1	0
