# Supplementary material for: Loss of function of 1-FEH IIb has more impact on post-harvest inulin degradation in Cichorium intybus than copy number variation of its close paralog 1-FEH IIa
Source: Front Plant Sci. 2015 Jun 23;6:455. doi: 10.3389/fpls.2015.00455 (PMC4477480; doi:10.3389/fpls.2015.00455)
Supplement: Supplementary file 3 [file Table_3.PDF]

Loss of function of 1-FEH IIb has more impact on post-harvest inulin degradation in *Cichorium intybus* than copy number variation of its close paralog 1-FEH IIa. Nicolas Dauchot[(*)] . Pierre Raulier . Olivier Maudoux . Christine Notté. Xavier Draye . Pierre Van Cutsem.
[(*)]Research Unit in Plant Biology, University of Namur, 61 rue de Bruxelles, 5000 Namur, Belgium e-mail: nicolas.dauchot@unamur.be
Frontiers in plant science

Supplementary table 3: For five parameters related with carbohydrate metabolism, proportion of the variance explained by an indel located in the 3'UTR of 1-FEH IIa and by the loss of mini-exon 2 in 1-FEH IIb. P values are not significant which indicates a strong correlation between both polymorphisms.

Target	DP		IN		SUCn		FFn		FGn	
	R^2	Pval	R^2	Pval	R^2	Pval	R^2	Pval	R^2	Pval
Indel 1FEH IIa	0,30	5,3E-09	0,42	8,2E-14	0,43	4,0E-14	0,31	2,2E-09	0,31	2,2E-09
Ex2 1FEH IIb	0,26	6,3E-08	0,44	2,4E-14	0,43	3,2E-14	0,32	5,3E-10	0,31	1,7E-09
