# Supplementary material for: Loss of function of 1-FEH IIb has more impact on post-harvest inulin degradation in Cichorium intybus than copy number variation of its close paralog 1-FEH IIa
Source: Front Plant Sci. 2015 Jun 23;6:455. doi: 10.3389/fpls.2015.00455 (PMC4477480; doi:10.3389/fpls.2015.00455)
Supplement: Supplementary file 4 [file Table_4.PDF]

**Loss of function of 1-FEH IIb has more impact on post-harvest inulin degradation in *Cichorium intybus* than copy number variation of its close paralog 1-FEH IIa.** Nicolas Dauchot(*) . Pierre Raulier . Olivier Maudoux . Christine Notté. Xavier Draye . Pierre Van Cutsem.
(*)Research Unit in Plant Biology, University of Namur, 61 rue de Bruxelles, 5000 Namur, Belgium
e-mail: nicolas.dauchot@unamur.be
Frontiers in plant science

**Supplementary table 4:** genotyping results obtained for 3 loci located in 1-FEH IIa and 1-FEH IIb on 112 individuals. Amplification products were scored on a ABi3130 XL fragment analyzer with 500-LIZ size standard. For each diploid locus, table presents the identified alleles that were scored. The first two columns are presenting the genotyping results of the 112 samples for the presence or absence of duplication in the 3' UTR of 1-FEH IIa. Absence of duplication is scored by a 427 bp long amplicon, while the presence of the duplication generates a 470 bp long fragment. Columns 3 to 6 corresponds to the peaks detected after analyzing the amplification products obtained with a single primer pair amplifying simultaneously part or the promotor of both 1-FEH IIa and 1-FEH IIb. Fragments 225 and 229 are amplicons resulting from the amplification of part of 1-FEH IIa promotor, while fragments of 247 and 302 bp long result from the amplification of 1-FEH IIb promotors. These results were used to propose the evolution of FEH II haplotypes (fig 4).

| | | F2a-indel | | F2a-prom | | F2b-prom | |
|---|---|---|---|---|---|---|---|
| | | 426 | 470 | 225 | 229 | 247 | 302 |
| | | | | | | | |
| Var_01 | X073 | 426,47 | 426,47 | 225,34 | | | 302,41 |
| Var_02 | X070 | 426,4 | 426,04 | 225,35 | | | 302,47 |
| Var_03 | X091 | 426,54 | 426,54 | 225,38 | | | 302,49 |
| Var_05 | X040 | 426,62 | 426,62 | 225,49 | | | 302,59 |
| Var_05 | X040 | 426,62 | 426,62 | 225,49 | | | 302,59 |
| Var_04 | X014 | 426,53 | 426,53 | 225,51 | | | 302,62 |
| L_02 | X191 | 426,59 | 426,59 | 225,51 | | | 302,64 |
| Var_01 | X080 | 426,48 | 426,48 | 225,53 | | | 302,47 |
| Var_06 | X018 | 426,36 | 426,36 | 225,55 | | | 302,66 |
| Var_04 | X013 | 426,39 | 426,39 | 225,59 | | | 302,56 |
| Var_08 | X172 | 426,44 | 426,44 | 225,68 | | | 302,77 |
| Var_08 | X164 | 426,7 | 426,7 | 225,67 | 229,42 | | 302,77 |
| L_01 | X195 | 426,5 | 426,5 | 225,53 | 229,35 | | 302,55 |
| Var_07 | X126 | 426,25 | 426,25 | 225,45 | 229,3 | | 302,46 |
| L_02 | X190 | 426,54 | 426,54 | 225,46 | 229,28 | | 302,54 |
| Var_01 | X076 | 426,52 | 426,52 | 225,5 | 229,28 | | 302,58 |
| Var_10 | X100 | 426,52 | 426,52 | 225,47 | 229,24 | | 302,48 |
| Var_01 | X083 | 426,51 | 426,51 | 225,48 | 229,24 | | 302,48 |
| Var_04 | X001 | 426,45 | 426,45 | 225,44 | 229,18 | | 302,85 |
| Var_11 | X137 | 426,58 | 426,58 | 225,38 | 229,16 | | 302,4 |
| Var_11 | X140 | 426,48 | 426,48 | 225,37 | 229,14 | | 302,48 |
| Var_07 | X128 | 426,53 | 426,53 | 225,38 | 229,09 | | 302,4 |
| Var_06 | X020 | 426,04 | 426,04 | | 229,28 | | 302,56 |
| Var_10 | X102 | 426,35 | 426,35 | | 229,26 | | 302,56 |
| Var_02 | X057 | 426,41 | 426,41 | | 229,23 | | 302,57 |
| Var_02 | X066 | 426,41 | 426,41 | | 229,04 | | 302,49 |
| L_01 | X197 | 426,43 | 426,43 | | 229,34 | | 302,63 |
| Var_07 | X129 | 426,47 | 426,47 | | 229,15 | | 302,49 |
| Var_10 | X104 | 426,48 | 426,48 | | 229,19 | | 302,05 |
| Var_10 | X104 | 426,48 | 426,48 | | 229,19 | | 302,05 |
| L_01 | X193 | 426,49 | 426,49 | | 229,36 | | 302,62 |
| Var_02 | X061 | 426,5 | 426,5 | | 229,26 | | 302,58 |
| Var_03 | X094 | 426,51 | 426,51 | | 229,26 | | 302,47 |
| Var_07 | X124 | 426,52 | 426,52 | | 229,13 | | 302,5 |
| Var_11 | X136 | 426,52 | 426,52 | | 229,08 | | 302,39 |
| Var_02 | X051 | 426,57 | 426,57 | | 229,02 | | 302,57 |
| Var_10 | X101 | 426,57 | 426,57 | | 229,15 | | 302,58 |
| Var_11 | X135 | 426,58 | 426,58 | | 229,15 | | 302,49 |
| L_01 | X194 | 426,58 | 426,58 | | 229,24 | | 302,62 |
| Var_02 | X067 | 426,52 | 470,93 | 225,39 | 229,08 | 247,62 | |
| Var_12 | X112 | 426,63 | 470,85 | 225,53 | 229,11 | 247,7 | 302,39 |
| Var_02 | X056 | 426,63 | 470,89 | 225,43 | 229,12 | 247,72 | 302,49 |
| Var_07 | X123 | 426,47 | 470,84 | 225,44 | 229,13 | 247,65 | 302,49 |
| Var_11 | X133 | 426,46 | 470,87 | 225,45 | 229,15 | 247,72 | 302,58 |
| Var_01 | X077 | 426,55 | 470,93 | 225,36 | 229,16 | 247,65 | 302,54 |
| Var_01 | X077 | 426,55 | 470,93 | 225,36 | 229,16 | 247,65 | 302,54 |
| Var_01 | X077 | 426,55 | 470,93 | 225,36 | 229,16 | 247,65 | 302,54 |
| Var_12 | X114 | 426,54 | 470,94 | 225,38 | 229,16 | 247,61 | 302,5 |
| Var_04 | X007 | 426,46 | 470,71 | 225,46 | 229,17 | 247,86 | 302,76 |
| Var_10 | X107 | 426,52 | 470,87 | 225,41 | 229,17 | 247,67 | 302,48 |
| Var_06 | X030 | 426,51 | 470,89 | 225,39 | 229,18 | 247,71 | 302,49 |
| Var_05 | X032 | 426,53 | 471,07 | 225,39 | 229,18 | 247,63 | 302,4 |
| Var_11 | X132 | 426,58 | 470,9 | 225,45 | 229,21 | 247,75 | 302,48 |
| Var_13 | X147 | 426,55 | 471,02 | 225,47 | 229,21 | 247,71 | 302,46 |
| Var_09 | X156 | 426,46 | 470,74 | 225,46 | 229,23 | 247,76 | 302,48 |
| Var_07 | X131 | 426,56 | 470,8 | 225,47 | 229,24 | 247,7 | 302,48 |
| Var_10 | X098 | 426,52 | 470,8 | 225,45 | 229,25 | 247,75 | 302,51 |
| Var_11 | X138 | 426,62 | 470,87 | 225,49 | 229,25 | 247,73 | 302,47 |
| Var_10 | X103 | 426,56 | 470,96 | 225,49 | 229,25 | 248,7 | 302,47 |
| Var_02 | X064 | 426,6 | 470,9 | 225,53 | 229,26 | 247,76 | 302,55 |
| Var_14 | X187 | 426,54 | 470,71 | 225,74 | | 248,08 | 302,83 |
| Var_08 | X169 | 426,44 | 470,77 | 225,68 | | 247,92 | 302,76 |
| Var_13 | X145 | 426,52 | 470,78 | 225,39 | | 247,75 | 302,49 |
| Var_04 | X012 | 426,61 | 470,8 | 225,62 | | 247,91 | 302,64 |
| Var_08 | X165 | 426,52 | 470,81 | 225,61 | | 247,87 | 302,7 |
| Var_01 | X078 | 426,5 | 470,96 | 225,38 | | 247,71 | 302,47 |
| Var_07 | X127 | 426,51 | 470,83 | 225,45 | | 247,73 | 302,48 |
| Var_06 | X027 | 426,53 | 470,85 | 225,46 | | 247,73 | 302,48 |
| Var_04 | X006 | 426,53 | 470,88 | 225,52 | | 247,8 | 302,56 |
| Var_01 | X081 | 426,45 | 470,87 | 225,4 | | 247,76 | 302,57 |
| Var_08 | X163 | 426,55 | 470,94 | 225,66 | | 248,03 | 302,78 |
| Var_09 | X154 | 426,53 | 470,81 | 225,45 | | 247,74 | |
| Var_14 | X186 | 426,49 | 471 | 225,65 | | 248,05 | |
| Var_09 | X158 | 426,68 | 471,01 | 225,44 | | 247,74 | |
| Var_14 | X189 | 426,59 | 471,08 | 225,56 | | 247,79 | |
| Var_12 | X120 | 470,55 | 470,55 | 225,38 | | 247,7 | |
| Var_15 | X173 | 470,67 | 470,67 | 225,65 | | 247,93 | |
| Var_04 | X003 | 470,69 | 470,69 | 225,52 | | 247,89 | |
| Var_02 | X065 | 470,74 | 470,74 | 225,21 | | 247,74 | |
| Var_03 | X087 | 470,78 | 470,78 | 225,43 | | 247,68 | |
| Var_03 | X087 | 470,78 | 470,78 | 225,43 | | 247,68 | |
| Var_03 | X087 | 470,78 | 470,78 | 225,43 | | 247,68 | |
| Var_12 | X118 | 470,78 | 470,78 | 225,41 | | 247,76 | |
| Var_15 | X174 | 470,78 | 470,78 | 225,7 | | 247,95 | |
| Var_15 | X179 | 470,78 | 470,78 | 225,57 | | 247,89 | |
| Var_05 | X046 | 470,8 | 470,8 | 225,45 | | 247,74 | 302,55 |
| Var_12 | X109 | 470,8 | 470,8 | 225,44 | | 247,74 | |
| Var_01 | X075 | 470,81 | 470,81 | 225,38 | | 247,72 | |
| Var_08 | X161 | 470,81 | 470,81 | 225,45 | | 247,77 | |
| Var_06 | X021 | 470,83 | 470,83 | 225,5 | | 247,77 | |
| Var_06 | X026 | 470,83 | 470,83 | 225,63 | | 247,89 | |
| Var_02 | X060 | 470,83 | 470,83 | 225,49 | | 247,72 | |
| Var_12 | X111 | 470,83 | 470,83 | 225,51 | | 247,75 | |
| Var_13 | X144 | 470,83 | 470,83 | 225,52 | | 247,79 | |
| Var_01 | X079 | 470,84 | 470,84 | 225,37 | | 247,77 | |
| Var_15 | X180 | 470,84 | 470,84 | 225,64 | | 248 | |
| Var_13 | X141 | 470,85 | 470,85 | 225,45 | | 247,75 | |
| Var_14 | X183 | 470,85 | 470,85 | 225,5 | | 247,85 | |
| Var_04 | X009 | 470,86 | 470,86 | 225,34 | | 248,52 | |
| Var_09 | X152 | 470,87 | 470,87 | 225,42 | | 247,78 | |
| V_06 | X024 | 470,88 | 470,88 | 225,46 | | 247,8 | |
| Var_01 | X072 | 470,89 | 470,89 | 225,42 | | 247,69 | |
| Var_15 | X175 | 470,89 | 470,89 | 225,7 | | 248,07 | |
| Var_05 | X041 | 470,91 | 470,91 | 225,48 | | 247,82 | |
| Var_12 | X119 | 470,91 | 470,91 | 225,49 | | 247,74 | |
| Var_15 | X177 | 470,91 | 470,91 | 225,64 | | 248 | |
| Var_01 | X082 | 470,94 | 470,94 | 225,46 | | 247,76 | |
| Var_04 | X015 | 470,96 | 470,96 | 225,55 | | 247,77 | |
| Var_15 | X181 | 470,96 | 470,96 | 225,55 | | 247,86 | |
| Var_05 | X036 | 470,97 | 470,97 | 225,42 | | 247,68 | |
| Var_05 | X034 | 470,99 | 470,99 | 225,46 | | 247,76 | |
| Var_08 | X162 | 470,99 | 470,99 | 225,43 | | 247,76 | |
