# Supplementary material for: Loss of function of 1-FEH IIb has more impact on post-harvest inulin degradation in Cichorium intybus than copy number variation of its close paralog 1-FEH IIa
Source: Front Plant Sci. 2015 Jun 23;6:455. doi: 10.3389/fpls.2015.00455 (PMC4477480; doi:10.3389/fpls.2015.00455)

Loss of function of 1-FEH IIb has more impact on post-harvest inulin degradation in *Cichorium intybus* than copy number variation of its close paralog 1-FEH IIa. Nicolas Dauchot(*) . Pierre Raulier . Olivier Maudoux . Christine Notté. Xavier Draye . Pierre Van Cutsem. (*)Research Unit in Plant Biology, University of Namur, 61 rue de Bruxelles, 5000 Namur, Belgium e-mail: nicolas.dauchot@unamur.be
Frontiers in plant science

Supplementary figure 1: Semi-quantitative RT-PCR analyses of fructan synthesis and degradation enzymes transcribed in chicory roots during growing season 1999-2000. Expression profiles correspond to 13 sampling dates for which respective minimal, average and maximal temperatures are presented. All the root tissues were sampled in field. RNA was normalized to a concentration of 1µg/µl. Tubulin was used as standard. As observed, 1-FFT transcription was constant during growing season. 1-SST expression started decreasing after exposure to temperatures lower than 4°C while, at the same time, 1-FEH IIb was over-expressed. 1-FEH IIb seemed to be expressed more specifically in response to exposure to cold temperatures, while 1-FEH I transcripts could be detected throughout growing season.

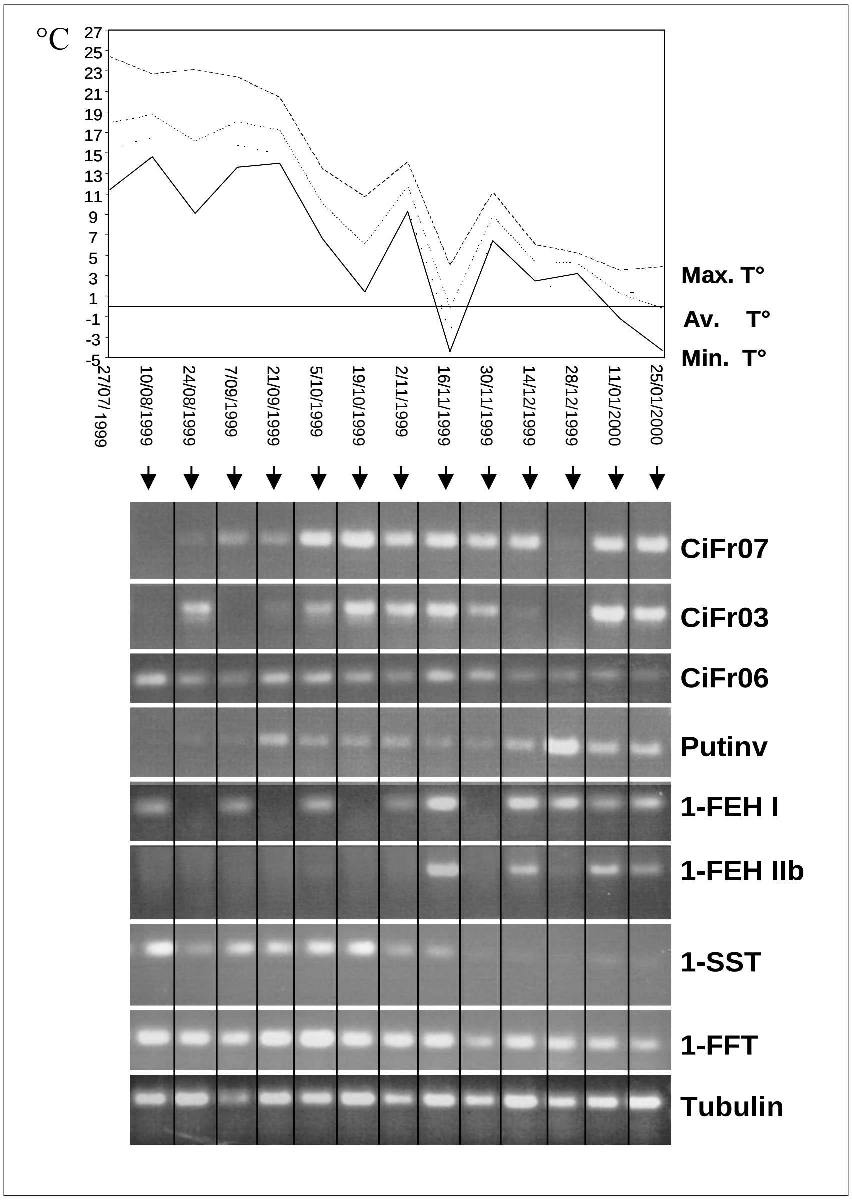

Supplement: Supplementary file 5 [file Image_1.PDF]
