# Supplementary material for: Loss of function of 1-FEH IIb has more impact on post-harvest inulin degradation in Cichorium intybus than copy number variation of its close paralog 1-FEH IIa
Source: Front Plant Sci. 2015 Jun 23;6:455. doi: 10.3389/fpls.2015.00455 (PMC4477480; doi:10.3389/fpls.2015.00455)
Supplement: Supplementary file 6 [file Image_2.PDF]

Loss of function of 1-FEH IIb has more impact on post-harvest inulin degradation in *Cichorium intybus* than copy number variation of its close paralog 1-FEH IIa. Nicolas Dauchot(*) . Pierre Raulier . Olivier Maudoux . Christine Notté. Xavier Draye . Pierre Van Cutsem.
(*)Research Unit in Plant Biology, University of Namur, 61 rue de Bruxelles, 5000 Namur, Belgium e-mail: nicolas.dauchot@unamur.be
Frontiers in plant science

Supplementary figure 2: Alignment of 1-FEH IIa putative orthologs retrieved after comparing AJ295033 against the NCBI EST database (BLASTn). 1-FEH IIa-1 *Helianthus annuus* is the contig of BU035623, GE492441, GE495456 and BU034394. 1-FEH IIa-2 *Helianthus annuus* is the contig of BU035062, BU031314, DY930605, DY921879, DY922023, DY955464 and BY03132. 1-FEH IIa *Lactuca sativa* is the contig of DW137787 and BQ844426. 1-FEH IIa tandem repeats are indicated by the two red boxes. This alignment indicates that the 47 bp duplication is absent from all publicly available 1-FEH II putative orthologs.

```
                                        1665                                                                                           .764
          1-FEH IIa - JQ585638 (R)   (93) TCATCTTTTTGTGTTTAATAATGGACTCAAAATGTCAAAATTTCGAAGATGAGTGCTTGGAGTATGAAGAATGCTAAATTTGTTGTTGATCAGAGTGTA
          1-FEH IIa - JQ585639 (S)   (93) TCATCTTTTTGTGTTTAATAATGGACTCAAAATGTCAAAATTTCGGAGATGAGTGCTTGGAGTATGAAGAATGCTAAATTTGTTGTTGATCAGAGTGTA
              1-FEH IIa - AJ295033 (1649) TCATCTTTTTGTGTTCAATAATGGACTCAAAATGTCAAAATTTCGGAGATGAGTGCTTGGAGTATGAAGAATGCTAAATTTGTTGTTGATCAGAGTGTA
              1-FEH IIb - AJ295034 (1632) TCATCTTTTTGCGTTTAATAACGGGACTCAAAATGTTAAAATTTCGAAGATGAGTGCTTGGAGTATGAAGAATGCTGCATTTGTTGTTGATCAGAATGTA
         EL369627-Cichorium endivia (142) TCATCTTTTTGCGTTTAATAATGGGACTCAAAATGTCAAAATTTCGGAGATGAGTGCTTGGAGTATGAAGAATGCTGAATTTGTTGTTGATCAGGGTGTA
       1-FEH IIa-1 Helianthus annuus  (2) TCATCTTTTTGCATTTAACAACGGCACTCAAAGCGTCAAGATTTCTCAAATGAGTGCTTGGAGTATGAAAAATGCGGAATTTACCATCGACCAAACCGTA
       1-FEH IIa-2 Helianthus annuus  (2) TCATCTTTTTGCATTTAACAACGGCACTCAAAGCGTCAAGATTTCTCAAATGAGTGCTTGGAGTATGAAAAATGCAGAATTTACCATCGGCCAAACCGTA
         EE643693-Helianthus exilis (393) TCATCTTTTTGCATTTAACAACGGCACTCAAAGCGTCAAGATTTCTCAAATGAGTGCTTGGAGCATGAAAAATGCGGAATTTACCATCGACCAAACCGTA
       EH724019-Centaurea maculosa (562) TCATCTTTTTGCTTTTAACAACGGGACTCGAAGCGTTACAATTTCGAAGATGAGTGCTTGGAGTATGAAGAATGCCGAATTTGTGATTGATCAGACCGTA
     DY839414-Taraxacum officinale (155) CCATCTTTTCGTTTTTAATAATGGTACTCAAGCTATCAAAATCTCTCAGATGAAGCTTGGAGCATGAAGAGTGCTGAATTTGTTGTGGACCAGACTGTA
          1-FEH IIa Lactuca sativa (230) CCACCTTTTCGCTTTTAATAATGGTACTCAAAGTGTCAAAATATCCCAAATGAGTGCTTGGAGTATGAAGAATGCTGAATTTGTTGTGGATCAGACTGTA
          DW046312-Lactuca saligna (107) CCACCTTTTCGCTTTTAATAATGGTACTCAAAGTGTCAAAATATCCCAAATGAGTGCTTGGAGTATGAAGAATGCTGAATTTGTTGTGGATCAGACTGTA
           DW164969-Lactuca virosa (131) CCACCTTTTCGCTTTTAATAATGGTACTCAAAGTGTCAAAATTTCCCAAATGAGTGCTTGGAGTATGAAGAATGCTAAATTTGTTGTGGATCAGACTGTA
                         Consensus (1665) TCATCTTTTTGC TTTAATAATGG ACTCAAAGTGTCAAAATTTC CAGATGAGTGCTTGGAGTATGAAGAATGCTGAATTTGTTGT GATCAGACTGTA
                                        1765                                                                                            1864
          1-FEH IIa - JQ585638 (R)  (193) AAAAGTGCAGCATAAAAAAAAT----------------------------------------------TAGAGGACAAGAATGTTAAAAATAGGTA-GAG
          1-FEH IIa - JQ585639 (S)  (193) AAAAGTGCAGCATAAAAAAAATAGAGGGCAAGAATGTTAAAAATGTAAAAAGTGCAGCATAAAAAAAATAGAGGGCAAGAATGTTAAAAATAGGTA-GAG
              1-FEH IIa - AJ295033 (1749) AAAAGTGCAGCATAAAAAAAATAGAGGGCAAGAATGTTAAAAATGTAAAAAGTGCAGCATAAAAAAAATAGAGGGCAAGAATGTTAAAAATAGGTA-GAG
              1-FEH IIb - AJ295034 (1732) AAAAGTGCAGCATAAAAATATTA---------------GAG-------------------------------GGGCTAGAATGTTAAAAATAGAT-----
         EL369627-Cichorium endivia (242) AAAAGTGCAGCATAAAAATATTAGAG----------------------------------------------GGGCTAGAATGTTAAAAATAGTTA-GAG
       1-FEH IIa-1 Helianthus annuus (102) AAAAGTGCAACTTAAAAATGCTA------------------------------------------------AGGGCTTGGATGTGTAAAATAGATATGAG
       1-FEH IIa-2 Helianthus annuus (102) AAAAGTGCAACTTAAAAATGCTA------------------------------------------------AGGGCTTGGATGTTTAAAATCGATACGAG
         EE643693-Helianthus exilis (493) AAAAGTGCAACTTAAAAATGCTA------------------------------------------------AGGGCTTGGATGTTTAAAATAGATATGAG
       EH724019-Centaurea maculosa (662) NAAAGTGCAGTTTAAGAAG--G-------------------------------------------------CA-TATATATACTGGAAACT--ATTAGAG
     DY839414-Taraxacum officinale (255) AAAAGCACAGTTTAAAAAAATA--------------TTAGAG----------------------------GAC-CTTAATATAT---AAATACGTA-AAT
          1-FEH IIa Lactuca sativa (330) AAAAGCACAATGTAAAAAA--TA-------------CTGAAG----------------------------GACACCGAATATATTTAAAAGAGGTA-GAT
          DW046312-Lactuca saligna (207) AAAAGCACAATGTAAAAAA--TA-------------TTGAGG----------------------------GACACCGAATATATTTAAAAGAGGTA-GAT
           DW164969-Lactuca virosa (231) AAAAGCACAATGTAAAAAA--TA-------------CTGAAG----------------------------GACCCCGAATATATTTAAAATAGGTA-GAT
                         Consensus (1765) AAAAGTGCAGC TAAAAAA  TA                                                GAGGGCTAG ATGTT AAAATAGGTA GAG
                                        1865                                                                                            1964
          1-FEH IIa - JQ585638 (R)  (246) TATTCTTGTTATAAAGTAAAGT------------------------------------------------------------------------------
          1-FEH IIa - JQ585639 (S)  (292) TATTCTTGTTATAAAGTAAAGT------------------------------------------------------------------------------
              1-FEH IIa - AJ295033 (1848) TATTCTTGTTATAAAGTAAAGTA-AAGGAACCAGTGAAGGTTGA-AAGAGTAGGTTTGGTAGCTCTTTCGTAAA-GATCATTCTTTGTTGCTG---TATT
              1-FEH IIb - AJ295034 (1781) -ATTCTTGTTATAAAGTAAAGTA-AAAGAAGCAGTGAAGGTCGA-AAGAGTAGGTTTGGTAGCTCTTTCATAAA-GATCATTCTTTGTTGCTT---TATT
         EL369627-Cichorium endivia (295) TATTCTTGTTATAAAGTAAAGTA-AAGGAAGCAGTGAAGGTCGA-AAGAGTAGGTTTGGTAGCTCTTTCATAAA-GATCATTCTTTGTTGCTT---TATT
       1-FEH IIa-1 Helianthus annuus (154) TATTCTTGTTTAAAGGTAAAGTAGAAC-AAACATTGGAGGCCGG-AAGAGTTGTTTAGATTTCTCTTTTGTAAAAGGTCATTCTTTGTTGCTTTAGTATT
       1-FEH IIa-2 Helianthus annuus (154) TATTCTTGTTTAAAGGTAAAGTAGAAC-AAACATTGGAGGCCGG-AAGAGTTGTTTAGATTTCTCTTTTGTAAA-GGTCATTCTTTGTTGCTTTAGTATT
         EE643693-Helianthus exilis (545) TATTCTTGTTTAAAGGTAAAGTAGAAC-AAACATTGGAGGCCGG-AAGAGTTGTTTAGATTTCTCTTTTGTAAAAGGTCATTCTTTGTTGCTTTAGTATT
       EH724019-Centaurea maculosa (708) TATTCTTGTGGAANAATATAGTA-AAAGAAACATTGGAGGCCATACAGAAGTGGTTTAATAGC-------------------------------------
     DY839414-Taraxacum officinale (309) TATTCTTATTGAA-----ATGTA-AAGGAAGCATCGGAGGCCGG-AAGAGTAGGTTTGGTAGCTCTTTCTTAGA-GGTCATTCTTTGTTGCAT---TATT
          1-FEH IIa Lactuca sativa (386) TATTCTTGTTAATTACT-AAATA-AAGGAAGCATTGGAGGCCGG-AAGAGTAGGTTTGTTAGCTCTTTCGTAGA-GGTCATTCTTTCTTGCTT---TATT
          DW046312-Lactuca saligna (263) TATTCTTGTTAATTACT-AAATA-AAGGAAGCATTGGAGGCCGG-AAGAGTAGGTTTGTTAGCTCTTTTATAGA-GGTCATTCTTTCTTGCTT---TATT
           DW164969-Lactuca virosa (287) TATTCTTGTTAATTACT-AAATA-AAGGAAGCATTGGAGGCCGG-GAGAGTAGGTTTGTTAGCTCTTTCGTAGA-GGTCATTCTTTCTTGCTT---TATT
                         Consensus (1865) TATTCTTGTTAAAAAGTAAAGTA AA GAA CATTGGAGGCCGG AAGAGTAGGTTTG TAGCTCTTT  TA A GGTCATTCTTTGTTGCTT   TATT
```
