# Supplementary material for: Loss of function of 1-FEH IIb has more impact on post-harvest inulin degradation in Cichorium intybus than copy number variation of its close paralog 1-FEH IIa
Source: Front Plant Sci. 2015 Jun 23;6:455. doi: 10.3389/fpls.2015.00455 (PMC4477480; doi:10.3389/fpls.2015.00455)
Supplement: Supplementary file 7 [file Image_3.PDF]

Loss of function of 1-FEH IIb has more impact on post-harvest inulin degradation in *Cichorium intybus* than copy number variation of its close paralog 1-FEH IIa. Nicolas Dauchot(*) . Pierre Raulier . Olivier Maudoux . Christine Notté. Xavier Draye . Pierre Van Cutsem. (*)Research Unit in Plant Biology, University of Namur, 61 rue de Bruxelles, 5000 Namur, Belgium e-mail: nicolas.dauchot@unamur.be

Frontiers in plant science

Supplementary figure 3: Alignment of the amino-acid sequence of the 3 known chicory FEH + the two newly isolated FEH (this paper), including the « wild type » long FEH-IIb and the mutated (short) FEH IIb allele, missing the mini-exon 2 (DPN residues). Positions of the 3 residues from the active site are highlighted by black boxes. Other mutations in the "short" FEH IIb allele are unlikely to restore the activity of this FEH allele.

```
                                1                                                                                         90
      1-FEH_I_CAC19366    (1) ----MCSERRVKEILGIWVLSLCLVWVQNGVGVHSSSPTEESQPYRTGFHFQPPKNWINDPNGPMYFNGVYHLFYQYNPYGPLWG-NISW
     1-FEH_IIa_AAP85536    (1) MKKSLSSFIVLCFLVIILETGRVKATSRNLNDVIMLANQQIEQPYRTGYHFQPPSNWMNDPNGPMLYQGVYHFFYQYNPYAATFGDVIIW
     1-FEH_IIb_CAC37923    (1) MKKSLSSFIALCFLVIVLETGRVKATSRDLNDVILLANQQIQQPYRTGYHFQPPSNWMNDPNGPMLYQGVYHFFYQYNPYAATFGDVIIW
1-FEH_IIb_long_AIP90173    (1) MKKSLSSFIALCFLVIVLETGRVKATSRDLNDVILLANQQIQQPYRTGYHFQPPSNWMNDPNGPMLYQGVYHFFYQYNPYAATFGDVIIW
1-FEHIIb_short_AIP90174    (1) MKKSLSSFIALCFLVIVLETGRVKATSRDLNDVILLANQQIQQPYRTGYHFQPPSNWMN---GPMLYQGVYHFFYQYNPYAATFGDVIIW
              Consensus    (1) MKKSLSSFIALCFLVIVLETGRVKATSRDLNDVILLANQQIQQPYRTGYHFQPPSNWMNDPNGPMLYQGVYHFFYQYNPYAATFGDVIIW
                                91                                                                                       180
      1-FEH_I_CAC19366   (86) GHSISYDLVNWFLLEPALSPKEPYDINGCLSGSATILPGPRPIILYTGQDVNNSQVQNLAFPKNLSDPLLKEWIKWSGNPLLTPVDDIKA
     1-FEH_IIa_AAP85536   (91) GHAVSYDLVNWIHLDPAIYPTQEADSKSCWSGSATILPGNIPAMLYTGSDSKSRQVQDLAWPKNLSDPFLREWVKHPKNPLITPPEGVKD
     1-FEH_IIb_CAC37923   (91) AHAVSYDLVNWIHLDPAIYPTQEADSKSCWSGSATILPGNIPAMLYTGSDSKSRQVQDLAWPKNLSDPFLREWVKHPKNPLIIPPEGVKD
1-FEH_IIb_long_AIP90173   (91) AHAVSYDLVNWIHLDPAIYPTQEADSKSCWSGSATILPGNIPAMLYTGSDSKSRQVQDLAWPKNLSDPFLREWVKHPKNPLIIPPEGVKD
1-FEHIIb_short_AIP90174   (88) AHAVSYDLVNWIHLDPAIYPTQEADSKSCWSGSATILPGNIPAMLYTGSDSKSRQVQDLAWPKNLSDPFLREWVKHPKNPLITPPEGVKD
              Consensus   (91) AHAVSYDLVNWIHLDPAIYPTQEADSKSCWSGSATILPGNIPAMLYTGSDSKSRQVQDLAWPKNLSDPFLREWVKHPKNPLITPPEGVKD
                                181                                                                                      270
      1-FEH_I_CAC19366  (176) GQFRDPSTAWMGPDGKWRIVIGSEIDGHGTALLYRSTNGTKWIRSKKPLHFSSKTGMWECPDFYPVTNGDKKGLDTSVQGNNTLHVLKVS
     1-FEH_IIa_AAP85536  (181) DCFRDPSTAWLGPDGVWRIVVGGDRDNNGMAFLYQSTDFVNWKRYDQPLSSADATGTWECPDFYPVPLNSTNGLDTSVYGGSVRHVMKAG
     1-FEH_IIb_CAC37923  (181) DCFRDPSTAWRGPDGVWRIVVGGDRDNNGMSLLYQSTDFVNWKRYDQPLSSAIATGTWECPDFYPVPLNSTNGLDTSVYGGSVRHVMKAG
1-FEH_IIb_long_AIP90173  (181) DCFRDPSTAWRGPDGVWRIVVGGDRDNNGMALLYQSTDFVNWKRYDQPLSSAIATGTWECPDFYPVPLNSTNGLDTSVYGGSVRHVMKAG
1-FEHIIb_short_AIP90174  (178) DCFRDPSTAWLGPDGVWRIVVGGDRDNNGMAFLYQSTDFVNWKRYDQPLSSADATGTWECPDFYPVPLNSTNGLDTSVYGGSVRHVMKAG
              Consensus  (181) DCFRDPSTAWLGPDGVWRIVVGGDRDNNGMALLYQSTDFVNWKRYDQPLSSA ATGTWECPDFYPVPLNSTNGLDTSVYGGSVRHVMKAG
                                271                                                                                      360
      1-FEH_I_CAC19366  (266) FNSREYYVIGTYDPIKDKFSVVT-NDFMVSNTQFQYDYGRYYASKSFYDSVNQRRVIWGWVNEGDSESDAVKKGWSGLQSFPRSIWLSNN
     1-FEH_IIa_AAP85536  (271) FEGHDWYTIGTYSPDRENFLPQNGLSLTGSTLDLRYDYGQFYASKSFFDDAKNRRVLWAWVPETDSQADDIEKGWAGLQSFPRALWIDRN
     1-FEH_IIb_CAC37923  (271) FEGHDWYTIGTYSPDRENFLPQNGLSLTGSTLDLRYNYGQFYASKSFFDDAKNRRVLWAWVPETDAPEDDIEKGWAGLQSFPRALWIDRS
1-FEH_IIb_long_AIP90173  (271) FEGHDWYTIGTYSPDRENFLPQNGLSLTGSTLDLRYDYGQFYASKSFFDDAKNRRVLWAWVPETDAPEDDIEKGWAGLQSFPRALWIDRS
1-FEHIIb_short_AIP90174  (268) FEGHDWYTIGTYSPDRENFLPQNGLSLTGSTLDLRYDYGQFYASKSFFDDAKNRRVLWAWVPETDAPEDDIEKGWAGLQSFPRALWIDRS
              Consensus  (271) FEGHDWYTIGTYSPDRENFLPQNGLSLTGSTLDLRYDYGQFYASKSFFDDAKNRRVLWAWVPETDAPEDDIEKGWAGLQSFPRALWIDRS
                                361                                                                                      450
      1-FEH_I_CAC19366  (355) RKQLVQWPVDEILKLRTKQVNITNRELAAGELLKIPSITASQADVEVSFSLTNLTEIELIDSEVVDPQLLCAQKNVSISGKFGPFGMLIL
     1-FEH_IIa_AAP85536  (361) GKQLIQWPVEEIEELRQNQVNLQNKNLKPGSVLEIHGIAASQADVTISFKLEGLKEAEVLDTTLVDPQALCNERGASSRGALGPFGLLAM
     1-FEH_IIb_CAC37923  (361) GKQLIQWPIEEIEELRQNEVNLQNKNLKPGSVLEIHGITASQADVTISFKLEELKDAEVLDTTSVDPQVLCNERGASSRGAIGPFGLLAM
1-FEH_IIb_long_AIP90173  (361) GKQLIQWPIEEIEELRQNEVNLQNKNLKPGSVLEIHGITASQADVTISFKLEELKDAEVLDTTSVDPQVLCNERGASSRGAIGPFGLLAM
1-FEHIIb_short_AIP90174  (358) GKQLIQWPVEEIEELRQNEVNLKNKNLKPGSVLEIHGITASQADVTISFKLEELKDAEVLDTTSVDPQVLCNERGASSRGAVGPFGLLAM
              Consensus  (361) GKQLIQWPVEEIEELRQNEVNLQNKNLKPGSVLEIHGITASQADVTISFKLEELKDAEVLDTTSVDPQVLCNERGASSRGAIGPFGLLAM
```

List of the differences present between AIP90173 and AIP90174

Deletion of 60-62 DPN residues in the sucrose binding box

174 I/T	both hydrophobic
191 R/L	charged + -> aliphatic
212 L/F	both hydrophobic
236 I/D	aliphatic -> charged - (too far from the active site to restore activity, at the periphery of the enzyme)
380 I/V	both aliphatic
384 K/Q	both polar -> + charged
443 I/V	both aliphatic
507 K/R	both polar + charged
577 N/S	both small polar
