# Supplementary material for: Loss of function of 1-FEH IIb has more impact on post-harvest inulin degradation in Cichorium intybus than copy number variation of its close paralog 1-FEH IIa
Source: Front Plant Sci. 2015 Jun 23;6:455. doi: 10.3389/fpls.2015.00455 (PMC4477480; doi:10.3389/fpls.2015.00455)
Supplement: Supplementary file 8 [file Image_4.PDF]

Loss of function of 1-FEH IIb has more impact on post-harvest inulin degradation in *Cichorium intybus* than copy number variation of its close paralog 1-FEH IIa. Nicolas Dauchot[(*)] . Pierre Raulier . Olivier Maudoux . Christine Notté. Xavier Draye . Pierre Van Cutsem.
[(*)]Research Unit in Plant Biology, University of Namur, 61 rue de Bruxelles, 5000 Namur, Belgium e-mail: nicolas.dauchot@unamur.be
Frontiers in plant science

Supplementary figure 4: At 55°C, primer pair F2b-82Fw and F2b-231Rv amplified both 150 bp long 1-FEH IIa cDNA and 1-FEH IIb allele missing the mini-exon 2 (scored as a 140 bp long fragment). cDNA was extracted from a chicory root collected in January 2012 after exposure to winter cold temperatures in a genotype homozygous for the 1-FEH IIb allele missing the mini-exon 2. This deleted allele is normally transcribed in the root. 1-FEH I was not amplified (expected 146 bp). Peak around 180 bp is an artefact due to the use of labelled dUTP during the PCR.

F2b-82Fw	CGGGATCTGAATGATGTGATACTA	(1-FEH IIb, AJ295034, 85-105)	left black box
F2b-231Rv	GTATTGGTAGAAGAAATGGTACACTCCT	(1-FEH IIb, AJ295034, 204-231)	right black box
Mini-exon	ATCCCAATG	(1-FEH IIb, AJ295034, 179-187)	red box

Expected amplification size 150 bp (full – 1-FEH IIb and 1-FEH IIa), 141 bp (1-FEH IIb without 9 bp mini-exon 2). PCR with FAM labelled dUTP.

Analyzed on ABi 3130Xl device with 500-Liz size standard and POP-4 polymer. Separation at 60°C.

```
                                1                                                                              80
1-FEH I    - AJ242538    (1)   GTTCTTTTTCTTTTCACGATCATTGGTGAGTTAATAAACCTGTCAGATCGACACTGAACAATGTGCT--CAGAAAGAAGG
 1-FEH IIa - AJ295033    (1)   ------------------------------------ACACACACACACTCATCATGAAGAAATCACTTTCTTCATTTATT
 1-FEH IIb - AJ295034    (1)   -----------------------------------------------------ATGAAGAAATCTCTTTCTTCATTTATT
             Consensus   (1)                                       A  C   CA A   A  ATGAAGAAATC CTTTCTTCATTTATT
                                81                                                                            160
1-FEH I    - AJ242538   (79)   GTAAAAGAGATACTTGGCATTTGGGTTCTCTCTCTTTGCTTAGTTTGGGTTCAGAATGGTGTTGGAGTTCATTCATCAT-
 1-FEH IIa - AJ295033   (45)   GTATTATGTTTTCTTGTCATCATTCTGGAAACTGGTCGGGTAAAA-GCGACCAGTC-GGAATCTGAATGATGTGATAATG
 1-FEH IIb - AJ295034   (28)   GCATTATGTTTTCTTGTCATCGTTCTGGAAACTGGTCGTGTAAAA-GCGACCAGTC-GGGATCTGAATGATGTGATACTA
             Consensus  (81)   GTATTATGTTTTCTTGTCATC TTCTGGAAACTGGTCG GTAAAA GCGACCAGTC GG ATCTGAATGATGTGATAAT
                                161                                                                           240
1-FEH I    - AJ242538  (158)   CTCCCA---CAGAAGAGTCG-CAGCCTTACAGGACTGGCTTCCACTTCCAGCCTCCTAAAAACTGGATAAACGATCCTAA
 1-FEH IIa - AJ295033  (123)   CTGGCGAATCAGCAGATTGAACAGCCGTATAGAACTGGATACCATTTTCAACCTCCAAGCAACTGGATGAACGATCCCAA
 1-FEH IIb - AJ295034  (106)   CTGGCGAATCAGCAGATTCAACAGCCGTATAGAACTGGATACCATTTTCAACCTCCAAGCAACTGGATGAACGATCCCAA
             Consensus (161)   CTGGCGAATCAGCAGATTCAACAGCCGTATAGAACTGGATACCATTTTCAACCTCCAAGCAACTGGATGAACGATCCCAA
                                241                                                                           320
1-FEH I    - AJ242538  (234)   CGGACCCATGTACTTCAATGGAGTTTACCATCTGTTCTACCAATACAACCCTTACGGTCCAC--TCTGGGG-CAACATTT
 1-FEH IIa - AJ295033  (203)   TGGACCAATGTTATACCAGGGAGTGTACCATTTCTTCTACCAATACAATCCGTATGCAGCAACGTTTGGTGACGTCATAA
 1-FEH IIb - AJ295034  (186)   TGGACCAATGTTATACCAAGGAGTGTACCATTTCTTCTACCAATACAATCCGTATGCAGCAACCTTTGGTGACGTCATCA
             Consensus (241)   TGGACCAATGTTATACCA GGAGTGTACCATTTCTTCTACCAATACAATCCGTATGCAGCAAC TTTGGTGACGTCAT A
```

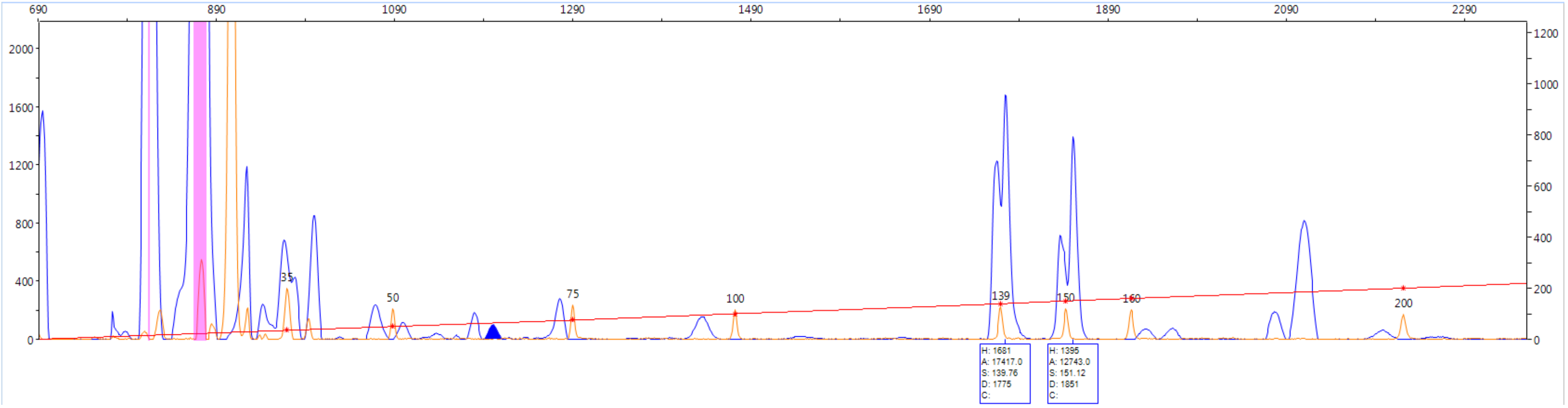

690
890
1090
1290
1490
1690
1890
2090
2290
2000
1600
1200
800
400
0
1200
1000
800
600
400
200
0
35
50
75
100
139
150
160
200
H: 1681
A: 17417.0
S: 139.76
D: 1775
C:
H: 1395
A: 12743.0
S: 151.12
D: 1851
C:
